# Advances and opportunities for computational interrogation of plant proteins

**DOI:** 10.1111/tpj.70899

**Published:** 2026-04-29

**Authors:** Sarah M. Bohling, Shaila Musharoff, Laura H. Gunn

**Affiliations:** ^1^ Plant Biology Section Cornell University Ithaca New York USA; ^2^ Department of Computational Biology Cornell University Ithaca New York USA

**Keywords:** computational plant biology, protein structure prediction, ancestral sequence reconstruction, molecular dynamics simulations, functional annotation, protein–protein interactions, post‐translational modifications, protein stability and flexibility, machine learning and protein modelling

## Abstract

Plants exhibit remarkable biochemical and physiological diversity, and are capable of adapting to a wide range of environmental conditions and stresses. This complexity makes them essential systems for understanding how life responds to a changing climate. Plant proteins are the molecular engines that carry out the reactions, signalling and regulation underlying these adaptive processes. However, studying plant proteins remains constrained by limited experimental throughput and the challenges of genetic manipulation, which vary widely across species. While synthetic biology and heterologous expression systems have expanded opportunities to investigate plant proteins, *in planta* studies are still limited by the availability and efficiency of genetic transformation methods. Computational approaches offer a powerful complement to experimental research by generating high‐throughput, testable hypotheses that can accelerate discovery of plant protein function. In recent years, the power, versatility and ease of use of computational tools for protein research have expanded dramatically. These methods now enable detailed predictions of protein structure, dynamics and interactions, as well as insights into their evolutionary history and mechanistic function. In this review, we highlight the expanding computational toolkit for plant protein analysis, emphasising both established and emerging approaches. We summarise recent successes where computational methods have provided key biological insights into plant protein function and highlight the potential of such methods for scientific discovery in plant research. By integrating computation with experimentation, plant biology can overcome current limitations to studying plant proteins and move more rapidly toward a mechanistic understanding of plant processes, enabling advances in agriculture, ecology and climate resilience.

## Significance Statement

Plant protein studies are often constrained by limited experimental throughput and transformation efficiency. Computational approaches provide a powerful complement, accelerating discovery, guiding hypothesis‐driven experiments and expanding the scope of questions that can be addressed in plant biology.

## INTRODUCTION

Plants are fundamental to life on Earth, removing carbon dioxide from the atmosphere through photosynthesis and generating the sugars that support nearly all life on this planet. Beyond nutrition, secondary plant metabolites used for medicines provide major benefits to human health (Eckardt, 2011). Plants are also essential for ecosystem health, as they contribute to climate regulation and provide raw materials for fibre and fuel (Dang et al., 2021). Due to these diverse and essential functions, understanding how plants function is critical for addressing pressing global challenges, from mitigating the impacts of climate change to improving agricultural sustainability and human health.

Plants are sessile: they cannot escape unfavourable environments or move toward favourable ones. Thus, they have evolved remarkable strategies to sense, respond to and adapt to stress. These adaptations span from rapid adjustments in protein stability and signalling networks to longer‐term modifications of gene expression, metabolism and morphology (for review see: Du et al., 2024). For example, plants can alter root architecture in response to soil salinity (Julkowska et al., 2017), produce protective compounds and osmoprotectants (Jahed et al., 2023) and synthesise heat‐shock proteins to preserve cellular function under stress (Mas‐ud et al., 2025). Understanding these strategies expands our knowledge of basic biology and offers opportunities for crop improvement and biotechnological advancement (Guo et al., 2020; Wu et al., 2024).

**Figure 1:**
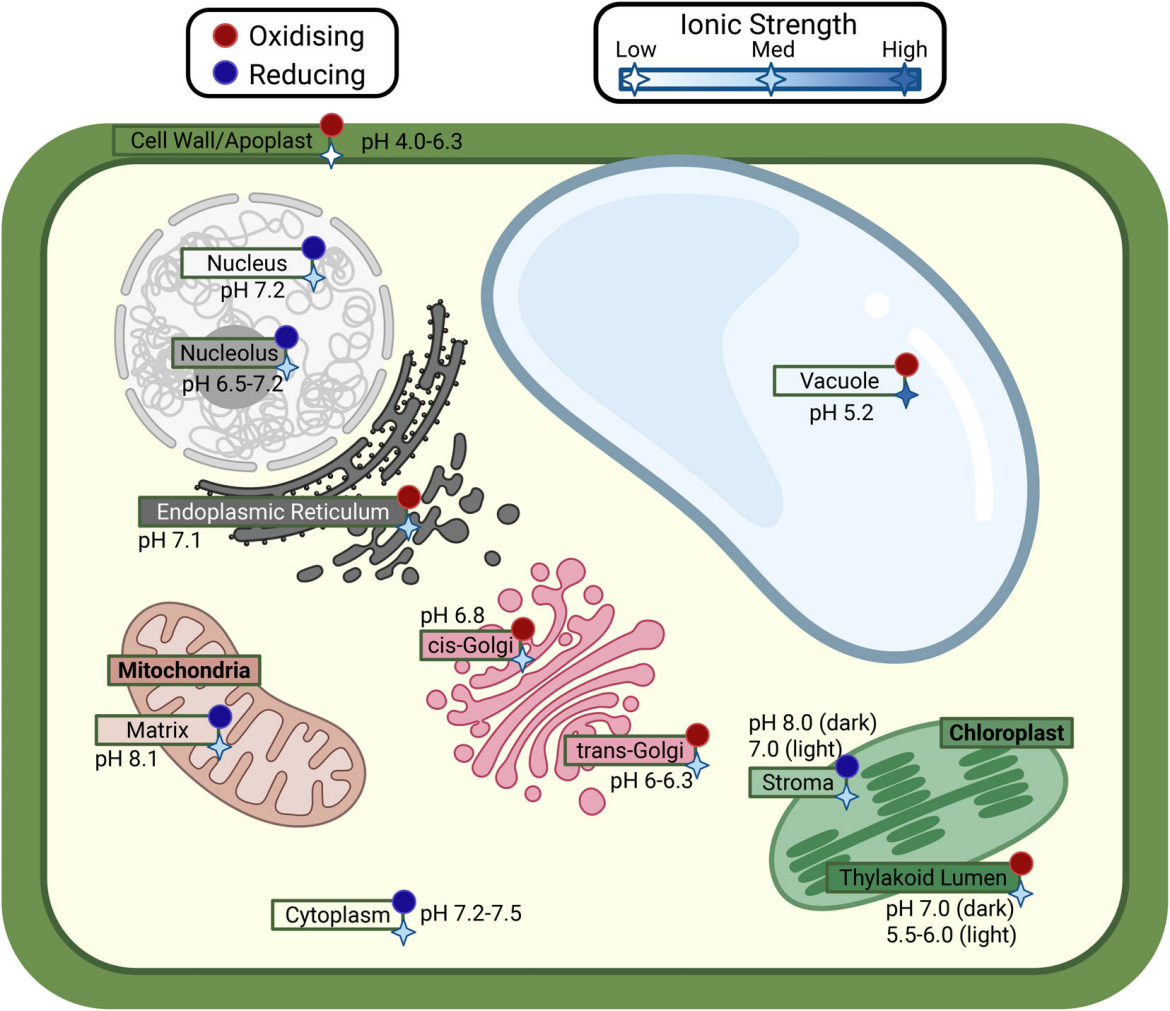
Genomic and physicochemical compartmentalization in plant cells. Schematic summarising typical pH, redox state and ionic strength across major plant compartments (nucleus, endoplasmic reticulum, cytosol, Golgi/TGN, chloroplast, mitochondrion, vacuole and apoplast). Protein‐coding genes in plants are distributed across three genetic compartments: the nuclear, chloroplast and mitochondrial genomes. Oxidising and reducing conditions are indicated by red and blue circles, respectively (Foyer & Noctor, 2013; Hanson et al., 2004; Hoh et al., 2024; Meyer et al., 2007; Miró‐Vinyals et al., 2025; Montacié et al., 2023; Queval et al., 2011; Schnaubelt et al., 2015). pH is shown as representative values or ranges (King et al., 2024; Kramer et al., 1999; Martinière et al., 2018; Shen et al., 2013; Werdan et al., 1975). Relative ionic strength is shown across compartments as low, medium or high as white, light blue and dark blue stars, respectively, with nucleolus ionic strength assumed to be roughly equivalent to that of the nucleus and cis‐golgi ionic strength assumed to be roughly equivalent to that of the trans‐golgi (for reference ion concentrations, see Portis & Heldt, 1976; Stein et al., 1979; Demmig & Gimmler, 1983; Robinson & Downton, 1984; Zottini & Zannoni, 1993; Fricke et al., 1994; Neuhaus & Wagner, 2000; Ishijima et al., 2003; Logan & Knight, 2003; Tester & Davenport, 2003; Carraretto et al., 2016; Szabò & Spetea, 2017; Raven, 2020; Hu et al., 2024). Note that compartments are dynamic, with chloroplast stroma and thylakoid lumen pH differing from day (light) to night (dark). Values vary with plant species, tissue, developmental stage and environment and are intended to provide a quick reference starting point for parameterizing environment‐aware computational analyses under plant‐relevant conditions. Created in BioRender. Bohling, S. (2026) https://BioRender.com/7ylkkw0.

**Figure 2:**
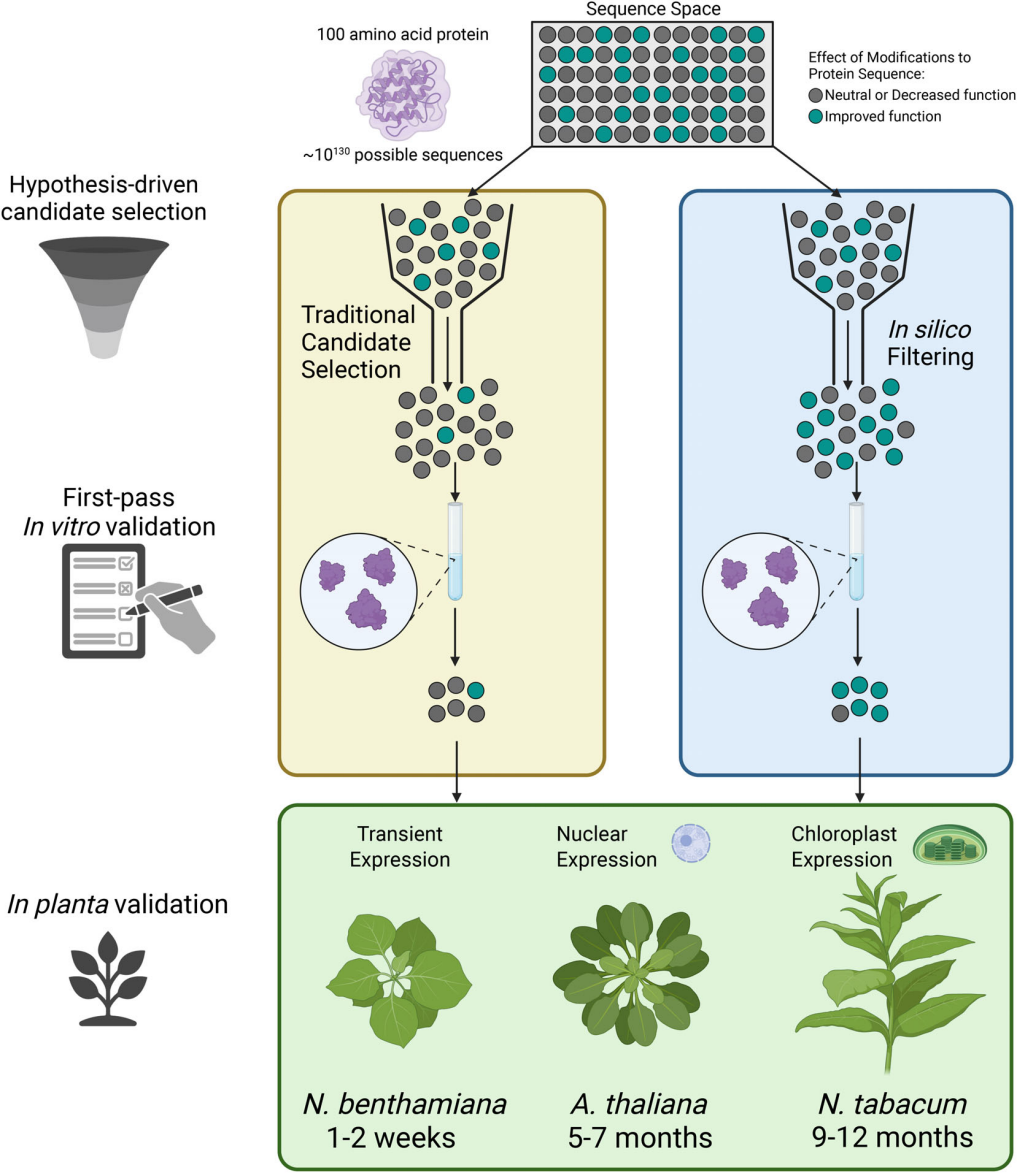
Computation narrows the experimental search space and accelerates descriptive‐to‐predictive discovery in plant biology. This figure illustrates a conceptual sequence space for a hypothetical 100 amino acid protein (≈10^130^ possible amino acid sequences). Each dot represents a candidate sequence variant; most variants are neutral or deleterious (grey), whereas a minority improve a desired property (teal). In the traditional workflow, knowledge‐guided prioritisation (e.g. literature‐ and structure‐informed hypotheses) provides an initial filtering step but typically advances a relatively unrefined candidate set into downstream testing. In the computation‐assisted workflow, *in silico* filtering (e.g. ancestral sequence reconstruction, structure/stability prediction, docking or saturation mutagenesis simulations) enables high‐throughput triage and yields a more selective funnel, enriching for improved candidates before experimentation. Candidates passing the filtering stage are evaluated in first‐pass *in vitro* assays, which provide an additional screen prior to plant experiments. Surviving candidates are then advanced to *in planta* testing using routes with distinct timelines, including transient expression (days to ~1–2 weeks from construct to readout), stable nuclear transformation in Arabidopsis (phenotypable T2 material typically ~3–4 months; homozygous T3 lines ~5–7 months), and chloroplast transformation in tobacco (often ~9–12 months to obtain phenotypable material). By prioritising candidates before time‐consuming plant experiments and reducing the number of constructs and lines that must be generated, computational filtering lowers the experimental burden while expanding the scale and scope of tractable plant biology questions. Created in BioRender. Bohling, S. (2026) https://BioRender.com/xrr5ol8.

Plant proteins carry out the biochemical reactions and regulatory processes that drive adaptation. However, studying plant proteins presents significant challenges. Many plant proteins are difficult to express in heterologous systems, in part due to complex folding requirements or extensive post‐translational modifications (PTMs). *In planta* studies face additional hurdles because plants encode proteins across nuclear, chloroplast and mitochondrial genomes (Figure 1), each with distinct limitations for genetic manipulation. Nuclear transformation is available for several model species but is generally additive, used to express transgenes rather than to introduce multiple targeted modifications and remains inefficient for precise genome editing (Wang, Si, et al., 2025). CRISPR technologies have expanded the plant genome modification toolkit, but editing efficiency varies by species and is typically limited in throughput (Gan & Ling, 2022). Chloroplast transformation is largely restricted to *Nicotiana tabacum* (tobacco) and remains labour‐intensive, while mitochondrial transformation remains poorly developed (Svab et al., 1990; Wang, Si, et al., 2025). Transient gene‐expression systems, such as those for *Nicotiana benthamiana*, enable rapid protein expression. However, their utility is limited to short‐term evaluation of protein localisation, interaction(s) or activity and are unsuitable for generating stable, heritable lines (Grützner et al., 2024). Due to these factors, experimental manipulation of plant proteins is often slow, costly and low‐throughput (Figure 2).

While model systems can substitute for direct genetic transformation, plants lack a single, broadly‐applicable experimental model. In human biology, fast‐growing model organisms with comparatively short generation times (e.g. *Caenorhabditis elegans*, *Mus musculus*) have enabled higher‐throughput experimental pipelines (e.g. Beraldo et al., 2019; Kerr et al., 2022). Analogous model systems exist in plant biology. For example, *Chlamydomonas reinhardtii* (Chlamydomonas), a unicellular model alga, has been used successfully to discover fundamental principles that extend to vascular plants. This system has been used to uncover the rules governing phase separation in pyrenoids (Freeman Rosenzweig et al., 2017), nuclear‐chloroplast gene‐expression coordination (Rochaix et al., 2014) and chloroplast chaperone and protease systems (Ramundo et al., 2014; Schroda et al., 2015). However, Chlamydomonas lacks key features of terrestrial plants including differentiated tissues and developmental gradients, and exhibits a vastly different lifestyle, mating cycle and cell wall composition compared to vascular plants. Thus, while Chlamydomonas is useful, it does not have universal utility as a plant model.

Specialised cell lines (e.g. CHO) have also accelerated scientific discovery in mammalian systems (Sharker & Rahman, 2021). An analogous specialised cell line in plant biology is tobacco BY‐2 suspension cells. Derived from pith tissue, these rapidly dividing and highly synchronised cells have been used to study the plant cell cycle, cytoskeletal dynamics and organelle inheritance (Nagata et al., 1992; Nebenführ et al., 2000; Seguié‐Simarro et al., 2004). They provide an opportunity for high‐throughput first‐pass screening for understanding fundamental plant cell processes and as an expression platform for plant proteins. Despite their experimental versatility and throughput, BY‐2 cells only partially capture the physiological and developmental complexity of plants.

Computational approaches offer exciting opportunities to study important plant proteins and processes, overcoming limitations to their study posed by few fast‐growing model systems and restricted transformation capability and speed. Advances in evolutionary analysis, protein structure prediction, molecular dynamics (MD) and network inference now enable researchers to study entire plant proteomes, pathways and processes, and to generate testable hypotheses at scale. By narrowing the experimental search space, these approaches can increase efficiency, reduce costs and accelerate the pace of discovery in plant biology (Figure 2).

In this review, we highlight successful applications of computational tools in plant biology, describe the underlying methods and recent innovations, discuss strategies for experimental validation, identify areas where these tools remain underutilised and outline future opportunities for integrating computation with experimentation to advance our understanding of fundamental plant processes.

## PROTEIN EVOLUTION AND ANCESTRY: ANCESTRAL SEQUENCE RECONSTRUCTION

Plants have a long evolutionary history, with major divisions (e.g. between angiosperms, gymnosperms and seedless plants) dating back hundreds of millions of years. Throughout evolutionary time, plant protein sequences, structures and functions have diversified as plants adapted to changing environments. Plant protein diversity has been influenced by myriad genetic changes including whole‐genome rearrangements and duplications, hybridisation, horizontal gene transfers and the unique evolutionary trajectories of chloroplast and mitochondrial genomes (One Thousand Plant Transcriptomes Initiative, 2019). Protein evolutionary histories can be computationally reconstructed to infer how protein function emerged and evolved. Ancestral sequence reconstruction (ASR) has been used to bridge the evolutionary past with present‐day biochemistry, generating diverse hypotheses for how plant proteins have adapted to changing environments over time and identifying reconstructed ancestral enzymes with increased activity, stability and promiscuity.

ASR predicts protein sequences at nodes of a phylogenetic tree. Extant protein sequences are aligned, a phylogenetic tree constructed and statistical models of sequence change applied to determine the most likely ancestral residue at each site. Evolutionary hypotheses from ASR can then be tested by experimentally characterising reconstructed proteins. Such methods produce inferences, not direct observations and thus their accuracy depends on the quality of the underlying data, alignments and models.

Several ASR frameworks exist, the two most common being maximum likelihood and Bayesian inference (for reviews see: Spence et al., 2021; Scossa & Fernie, 2021; Thomson et al., 2022). Maximum likelihood, currently the most widely used framework, identifies the most probable ancestral sequence by calculating the probability of observing the extant sequences given each possible ancestral sequence. Maximum likelihood incorporates a substitution rate matrix that accounts for unequal amino acid substitution rates. While classical maximum likelihood approaches do not explicitly account for uncertainty in the alignment, phylogeny or chosen substitution model, modern workflows have made great progress in mitigating this limitation by integrating phylogenetic tools. For example, IQ‐TREE uses an efficient stochastic/heuristic tree‐search strategy to explore phylogenetic space from an input alignment, and identify the highest‐likelihood topology (Nguyen et al., 2015). IQ‐TREE now also incorporates ModelFinder, which evaluates candidate substitution models and identifies the model of evolution best supported by the alignment data, thereby reducing substitution model uncertainty in ASR pipelines (Kalyaanamoorthy et al., 2017; Wong et al., 2025). Alignment uncertainty can likewise influence reconstruction accuracy, and incorporating multiple alternative alignments into maximum likelihood ASR has been shown to reduce overall reconstruction error compared to using a single input alignment (Aadland & Kolaczkowski, 2020).

Bayesian inference directly incorporates measures of uncertainty by sampling distributions of tree topologies, branch lengths and evolution model parameters (Hanson‐Smith et al., 2010; Huelsenbeck & Bollback, 2001; Pagel et al., 2004; Schultz & Churchill, 1999). The primary drawback of Bayesian inference is computational cost, and its application is thus typically limited to smaller datasets. While Bayesian inference explicitly quantifies and propagates uncertainty in ancestral reconstructions, maximum‐likelihood approaches can be equally or even more accurate, depending on the input alignment, phylogeny and substitution model. When tested on simulated data, maximum likelihood and Bayesian inference were found to have accuracies of ~94% and ~92%, respectively (Williams et al., 2006). These accuracy figures reflect performance on simulated datasets under specific conditions and do not capture Bayesian inference's distinct advantage in uncertainty quantification (e.g. posterior probabilities over trees, model parameters and ancestral states).

Recent advances in ASR integrate structural biology and incorporate machine learning (ML) into high‐throughput computational workflows to enhance prediction accuracy and reduce biases such as systematic overestimation of thermostability (Thomson et al., 2022). They also increase the ease of adoption of ASR methods by researchers without extensive computational expertise.

Structure‐aware approaches combine evolutionary and biophysical information to enhance inference. Classical ASR methods treat residues as independent sites in a sequence, with substitution probabilities informed solely by substitution models. Reconstructions become more realistic when the structural context is taken into account, such as whether a residue is solvent‐exposed or buried (Moshe & Pupko, 2019). Tools such as ProtASR (Arenas et al., 2017) account for folding stability. New substitution matrices such as RAM55 incorporate rotamer geometries and outperform sequence‐only models to reconstruct functionally plausible ancestral proteins (Perron et al., 2019).

Neural network‐based methods are now being used for sequence alignments (e.g. BetaAlign, Dotan et al., 2025), phylogenetic tree inference (e.g. Phyloformer, Nesterenko et al., 2025) and to evaluate ASR‐derived enzyme libraries to predict features like improved thermostability (Brennan et al., 2024). As protein language models continue to improve, they will likely be increasingly coupled with ASR to predict ancestral sequences that better capture epistatic constraints (the coupled effects of mutations at different residues on protein structure and function) and structure–function relationships.

Method accessibility has been improved through increased availability of automated web servers and pipelines. Resources including FastML (Ashkenazy et al., 2012), FireProtASR (Musil et al., 2021) and ProtASR2 (Arenas & Bastolla, 2020) enable users to perform ASR with minimal computational expertise, often requiring only an input sequence or alignment. The increasing number of user‐friendly tools lowers barriers to adoption of ASR across fields. Complementing these resources, the Revenant database (Carletti et al., 2020) provides a curated repository of reconstructed ancestral proteins, which, although not comprehensive, represents a step toward community‐accessible reference data.

For reconstructions of ancestral chloroplast‐encoded plant protein sequences, chloroplast‐specific models must be used to model protein evolution. Chloroplasts are central to plant metabolism and photosynthesis. Of the roughly 3000 proteins that function within the chloroplast, only about 80–100 are encoded by the plastid genome. Yet, these include many of the core components essential for photosynthetic energy conversion and gene expression, such as subunits of photosystems I and II, Rubisco, ATP synthase and the plastid ribosome (Long et al., 2022; Rossig et al., 2013; Xing et al., 2022). Several chloroplast‐specific models have been developed, and the most commonly used chloroplast model, cpREV, has been incorporated into existing ASR programs (Adachi et al., 2000). Yet, despite intensive study of plastid‐encoded proteins in green plants, gcpREV, a Viridiplantae‐specific model, remains heavily underutilised in phylogenetic and ASR analyses (Brazão et al., 2023; Cox & Foster, 2013).

In plant biology, ASR has been used to study how stability and specificity evolved in plant protein families. Two notable ASR studies on (i) the thermostability of green plant Nucleoside diphosphate kinases (Garcia et al., 2017) and (ii) the enzymatic activity and reaction enthalpies of Solanaceae Rubiscos (Lin et al., 2022) revealed trends that correlate with Earth's heating and cooling over geological time. Reconstructed ancestral peroxidases from *Glycine max* (soybean) or horseradish lineages were found to display greater stability (Loughran et al., 2014) and reconstructed ancestral hydroxynitrile lyase was found to exhibit a higher melting temperature than modern homologues (Devamani et al., 2016). Similarly, reconstructed ancestral iridoid synthase and hydroxynitrile lyase were found to exhibit higher promiscuity (i.e. expanded specificity) than extant enzymes (Devamani et al., 2016; Lichman et al., 2020).

Reconstructed ancestral proteins can also provide insights into molecular function. For example, ASR of chalcone isomerase suggests that its catalytic function in flavonoid biosynthesis arose from duplication of a noncatalytic ancestor, followed by substitutions forming the flavanone‐binding pocket (Kaltenbach et al., 2018). ASR analysis of EPYC1, an intrinsically disordered pyrenoid linker protein, suggests that all ancestral EPYC1 could induce phase separation (Küffner et al., 2024). Additional studies have traced plant Rubisco's evolutionary trajectory to determine that its chaperone dependency evolved neutrally, not adaptively (Ng et al., 2025). Furthermore, ancestral reconstructed proteins tend to exhibit increased thermostability, activity and substrate tolerance relative to extant proteins (Furukawa et al., 2020; Thomson et al., 2022), and thus can be a valuable method for engineering more stable, active and promiscuous enzymes (for review, see Spence et al., 2021).

Because ancestral sequences cannot be directly observed, validation of ASR workflows has traditionally relied on benchmarking with simulated datasets. New approaches such as extant sequence reconstruction (ESR) (Sennett & Theobald, 2024) provide more empirical measures of accuracy, allowing researchers to test pipelines by attempting to reconstruct known, modern protein sequences. Ultimately, the most robust validation comes from experimental characterisation of reconstructed proteins. Functional testing via heterologous or *in planta* expression of ancestral proteins enables direct assessment of solubility, stability, activity and specificity to confirm whether predicted properties align with computational expectations.

Recent advances are transforming ASR from a specialist technique into a versatile and increasingly mainstream tool. Integration with ML and protein language models can reduce uncertainty by capturing epistatic constraints, improving alignments and predicting plausible ancestral variants. More sophisticated species‐aware phylogenomic models will help accommodate hybridisation and genome duplication, while structure‐aware models will improve accuracy for organelle proteins. While ASR has been used to trace the evolutionary trajectories of plant protein function and discover enzyme variants with beneficial properties, its potential in plant biology remains far from fully realised. The evolutionary history of many plant protein families is still poorly understood, and plant pathway engineering strategies have yet to fully exploit ASR to design enzymes with improved thermostability, activity or promiscuity. Expanding databases such as Revenant, and integrating plant‐specific data will improve availability of reconstructed proteins. Integration of green plant chloroplast‐specific models (e.g. gcpREV) into ASR workflows and broader application of ASR to new plant lineages and pathways offers immense opportunity to uncover evolutionary mechanisms and test mechanistic hypotheses about plant protein function.

## FUNCTION, LOCALISATION AND MODIFICATION

Modern plant biology faces an abundance of omics data but a relative scarcity of experimentally‐validated protein functions. Computational methods integrate sequence information, ML and experimental data to infer function, localisation and post‐translational regulation, providing context for interpreting omics datasets, reconstructing signalling pathways and prioritising targets for experimental validation. For plant proteins, such approaches are essential to navigate the characteristic large gene families, organelle‐specific targeting and complex environmental regulatory mechanisms.

### Functional annotation

Functional annotation methods assign biological function to uncharacterised proteins. In plants, annotation is complicated by lineage‐specific diversification and the presence of more than one organellar genome. While homology‐based methods can often infer function in well‐studied model plants, these approaches are limited when proteins lack close homologues or belong to rapidly evolving or duplicated families. Thus, functional annotation methods increasingly integrate comparative genomics, variant‐effect prediction and ML to predict biochemical roles.

Functional annotation methods can be grouped into three categories: variant‐based, sequence‐based and ML‐based. Variant‐based tools such as SnpEff (Cingolani et al., 2012) and ANNOVAR (Wang et al., 2010) predict the functional consequences of nucleotide substitutions, insertions or deletions. Downstream tools like MAGMA (de Leeuw et al., 2015) can then link variant sets to genes and pathways, enabling mechanistic interpretations of complex phenotypes. Sequence‐based tools rely on evolutionary conservation to directly infer function from sequence. Classical examples include SIFT (Kumar et al., 2009) and PolyPhen‐2 (Adzhubei et al., 2010), which estimate the effect of substitutions on stability and activity. These methods are computationally efficient and easily implemented, but depend heavily on the availability of high‐quality homologous sequences, which are often sparse for non‐model plants.

ML‐based methods are transforming functional annotation by integrating evolutionary and structural information. Deep‐learning models are increasingly being developed for direct protein function prediction from sequence and/or structure, and can improve performance when close homologues are lacking. For example, DeepGOPlus predicts ontology classes for protein sequences using sequence similarity and deep learning (Kulmanov & Hoehndorf, 2020). DeepFRI leverages protein language model features with structural information to infer function and highlight functionally important residues (Gligorijević et al., 2021). DeepECTransformer, a bacteria‐specific deep neural network method, predicts enzyme commission numbers from sequence embeddings (Kim et al., 2023). These frameworks, when combined with protein databases such as UniProt, Pfam and InterPro, can classify proteins into functional categories in the absence of homologues and provide natural starting points for plant‐focused retraining or transfer learning as experimentally supported plant annotations expand.

Functional annotation in plant biology is moving toward more integrated, species‐agnostic and plant‐trained models. Most existing tools are optimised for animal or microbial genomes and often fail to account for plant‐specific features, such as codon bias, polyploidy and regulatory sequence patterns. ML frameworks can be retrained on plant genomic datasets and to incorporate features from omics layers, including expression and localisation data. Similarly, human‐specific tools like OpenCRAVAT (Pagel et al., 2020) and existing pipelines combining annotation tools (e.g. WGSA, Liu et al., 2016) could be modified to support comparative annotation across crop lineages.

While most existing functional annotation approaches are not plant‐specific, several commonly used annotation tools do incorporate plant genomes. For example, SIFT includes databases for 18 plant species such as *Zea mays* (maize), *Arabidopsis thaliana* (Arabidopsis), *Brassica rapa*, soybean and *Sorghum bicolor* (Kumar et al., 2009). Such tools have been used in plant biology for GWAS and to predict deleterious variants and candidate genes contributing to agronomically relevant phenotypes. SIFT was used in combination with additional annotation tools to predict deleterious variants that could impact climate adaptation in Arabidopsis populations (Jiang et al., 2025). Higher‐throughput examples of predicting trait variation using ANNOVAR include identifying variants contributing to ethylene insensitivity in Arabidopsis (Wang, Luo, et al., 2025) and differences in function of analogous genes across species of buckwheat (Shi et al., 2025). GWAS pipelines, such as MASH (Urbut et al., 2019), have mapped traits in sorghum and *Panicum virgatum*, identifying loci linked to sugar metabolism, biomass allocation and panicle architecture (Boatwright, Sapkota, Jin, et al., 2022; Boatwright, Sapkota, Myers, et al., 2022; Zhang, MacQueen, et al., 2022).

While functional annotation dramatically accelerates hypothesis generation, experimental validation of predicted functions remains essential, bridging plant genotypes and phenotypes. Predicted protein function can be experimentally validated using biochemical, genetics and omics approaches. For example, CRISPR/Cas‐mediated knockouts (or RNAi knockdowns) can directly test whether loss of a predicted activity produces the anticipated developmental, physiological or stress‐response phenotype. For enzymatic predictions, the most direct validation of predicted function is targeted biochemical assays, which enable *in vitro* confirmation of substrate specificity and kinetic properties. To further refine computationally‐predicted protein functions and prioritise mechanistic follow‐up, plant pipelines are increasingly combining perturbation genetics with multi‐omics readouts to identify downstream pathway signatures that can help place previously uncharacterised proteins into regulatory or metabolic networks (Henchiri et al., 2024; Montes et al., 2022).

### Post‐translational modifications

PTMs expand the functional diversity of proteomes, acting as molecular switches to control enzyme activity, signal transduction and subcellular fate. For plants, PTMs enable rapid, reversible adaptation to environmental fluctuations such as drought, temperature and pathogen attack (Li & Liu, 2021). Computational PTM predictors can identify candidate regulatory sites and map signalling networks that are challenging to experimentally trace. Among the thousands of known PTMs, phosphorylation, glycosylation and ubiquitination dominate plant biology (for reviews see: Li & Liu, 2021; Strasser et al., 2021; Wang, Liu, et al., 2024).

Phosphorylation, glycosylation and ubiquitination predictors commonly use ML and deep‐learning algorithms, trained on experimentally‐validated datasets, to identify modification sites based on sequence, structure, evolutionary conservation and biophysical features.

Phosphorylation, the covalent addition of a phosphate (typically to serine, threonine or tyrosine), alters local charge and conformation, enabling proteins to switch between active and inactive states. In plants, phosphorylation underlies nearly all major signalling cascades, including those for abscisic acid, drought and pathogen response (Li & Liu, 2021). Prediction platforms such as Phosformer (Zhou, Yeung, et al., 2023) and MusiteDeep (Wang et al., 2020) use ML methods to identify likely phosphorylation sites. Plant‐specific PTM databases, such as P^3^DB (Yao et al., 2014) and PhosPhAt (Durek et al., 2010), provide experimental data from model species, which can be used for model training and benchmarking.

Glycosylation is the covalent attachment of carbohydrates, typically to asparagine (N‐linked) or serine/threonine (O‐linked), and is involved in regulating immune responses, pollen development and hormone signalling, as well as the folding, trafficking and stability of membrane and secreted proteins (Strasser et al., 2021). Unlike in animals, plant O‐glycosylation can occur on hydroxyproline residues within extensins and arabinogalactan proteins (Kieliszewski, 2001). Common prediction methods include StackGlyEmbed (Nafi & Rahman, 2025) and DeepNGlyPred (Pakhrin et al., 2021), which leverage ML or protein language models to identify glycosylation sites in eukaryotic and human proteomes with improved accuracy.

Ubiquitination, the attachment of a 76‐amino acid ubiquitin polypeptide to lysine, serine or cysteine residues, regulates protein turnover and signal transduction (Komander & Rape, 2012). In plants, ubiquitination is involved in a range of processes, including hormone signalling, defence responses and photomorphogenesis (Ma et al., 2020; Wang, Liu, et al., 2024). Predictors such as UbPred (Radivojac et al., 2010) and the plant‐specific method UPFPSR (Yin et al., 2022) use ML methods, including ensemble and deep learning, to identify ubiquitin‐acceptor residues.

Recent innovations in PTM prediction have expanded the scope and precision of plant proteome analysis. Recent tools, including StackGlyEmbed (Nafi & Rahman, 2025), EMNGly (Hou et al., 2023) and Phosformer (Zhou, Yeung, et al., 2023), leverage deep‐learning approaches and transfer learning from large protein language models to improve prediction accuracy across species. Such methods can extend PTM analysis beyond histones to the broader plant proteome by integrating sequence, structural and PTM co‐occurrence information to identify candidate sites that may regulate enzyme activity, metabolism and stress adaptation.

Computational tools for predicting PTMs have been increasingly used to investigate protein regulation, signalling and stress responses in plants. Phosphorylation predictors, such as MusiteDeep, have been used across multiple plant species including Arabidopsis, *Brassica napus* and maize (Yao et al., 2012). MusiteDeep, an Arabidopsis‐trained model, could accurately identify phosphorylation sites in other green plants, suggesting that deep‐learning PTM predictors are somewhat generalisable among plants. NetNGlyc (Gupta & Brunak, 2002), a glycosylation predictor, has successfully identified specific N‐glycosylation sites in the *N. benthamiana* Kunitz peptidase inhibitor‐like protein (KPILP), despite being originally developed for human proteins (Ershova et al., 2022). Ubiquitination predictors have identified regulatory mechanisms in crop and model species. For example, UbPred was used to predict lysine residues in the *Oryza sativa* (rice) flowering repressor Ghd7 that undergoes sucrose‐induced polyubiquitination, targeting the protein for degradation (Cho et al., 2024). Ubiquitination prediction tools have also been used to identify ubiquitinated lysines in Arabidopsis MAPKKK18, which regulates abscisic acid signalling and drought tolerance (Tajdel‐Zielińska et al., 2023).

While computational predictions dramatically accelerate hypothesis generation, experimental validation remains necessary. Mass Spectrometry (MS) is the gold standard to identify PTM sites, complemented by enrichment strategies such as immobilised metal affinity chromatography for phosphopeptides (Mann et al., 2002; Ruprecht et al., 2017) or lectin affinity assays for glycoproteins (Goumenou et al., 2021). Antibody‐based approaches, including phospho‐specific or anti‐diGly immunoblots, can validate modification state and turnover dynamics. Emerging plant proteomics pipelines combine TurboID proximity labelling with liquid chromatography–tandem mass spectrometry (LC–MS/MS) to map PTM networks *in vivo* (for review of TurboID in plants, see Feng et al., 2024; Park & Kim, 2025).

### Localisation prediction connects molecular function to cellular context

The cellular context a protein operates in determines its access to substrates, cofactors and interaction partners, all of which define a protein's physiological function. For example, chloroplast‐targeted enzymes function in distinct redox, pH and ionic conditions, while cytosolic paralogues may participate in related reactions under entirely different constraints (Figure 1). Subcellular localisation can also suggest broad functions: for example, given that photosynthesis occurs in the chloroplast, chloroplast localisation suggests a photosynthetic function. Localisation prediction requires consideration of the compartmental complexity of plant cells. Nuclear‐encoded chloroplast and mitochondrial proteins are synthesised in the cytosol and directed to their destination by N‐terminal transit peptides. Proteins can also localise to peroxisomes and vacuoles through signals at either terminus. Moreover, dual targeting and relocalisation are common: proteins may be directed to both chloroplasts and mitochondria or shuttled between the nucleus and cytoplasm in response to stress or developmental cues. Predictive algorithms must therefore capture context‐dependent flexibility.

Early localisation prediction tools, such as WoLF PSORT (Horton et al., 2007) relied primarily on amino acid composition, charge and sequence motifs to infer a protein's subcellular location. In these models, certain biophysical features, such as hydrophobic signal peptides or basic transit sequences, are statistically linked to known compartments. While useful, these approaches were constrained by the quality and breadth of the training data. For example, many were trained largely on land‐plant datasets and thus struggled to identify targeting sequences in other photosynthetic eukaryotes, as organelles can differ in origin and membrane complexity: plant chloroplasts arose from a single primary endosymbiotic event, whereas diatoms and many algae experienced secondary or tertiary endosymbioses, resulting in additional membrane layers surrounding their plastids (Nisbet et al., 2004).

Recent tools integrate ML to improve both accuracy and coverage across a variety of lineages, including plants. For example, DeepLoc 2.1 (Ødum et al., 2024) uses ML with protein language models to identify sorting signals including chloroplast‐targeting peptides. Similarly, LocPro (Zhang, Zheng, et al., 2025) uses a hybrid convolutional, fully connected and bidirectional neural network to allow localisation assignment to single or multiple compartments. These advances make it possible to predict localisation for entire plant proteomes with greater precision and throughput.

Emerging innovations are blurring the line between prediction and observation. Deep generative models, such as deepGPS (Yuan et al., 2025), and protein language model‐based approaches like PUPS (Zhang, Tseo, et al., 2025), combine protein sequence and fluorescence‐image data to infer spatial distribution within human cells. Localisation predictors trained on plant‐specific microscopy datasets could automatically annotate subcellular locations across plant proteomes. Furthermore, integration with structural prediction tools and interactome‐mapping algorithms has the potential to resolve multi‐component assemblies within compartments such as chloroplasts, thylakoids and peroxisomes.

Plant‐specific localisation prediction tools have already proven valuable. LOCALIZER (Sperschneider et al., 2017) detects nuclear localisation signals and transit peptides targeting chloroplasts and mitochondria, outperforming general eukaryotic models for plant sequences. Plant‐mSubP (Sahu et al., 2020) is a ML‐based method trained on UniProt‐annotated plant proteins, allowing single and multiple localisation assignment of proteins to more than 10 subcellular compartments. Both have been validated across diverse taxa. For instance, LOCALIZER correctly identified the nuclear localisation of the oxidative‐stress regulator PpNBS1 in *Physcomitrium patens* (Das et al., 2025) and the chloroplast targeting of the terpenoid‐biosynthetic enzyme McDXS in *Monarda citriodora* (Sharma et al., 2025), while Plant‐mSubP has been used to study disease resistance in cassava and melatonin‐receptor localisation in *Capsicum chinense* (Phaisomboon et al., 2025; Toledo‐Castiñeira et al., 2025).

Localisation predictors are especially useful in large‐scale plant genomic or transcriptomic studies to narrow down candidate proteins from large expression datasets. For example, these approaches have been used to identify and characterise members of the DnaJ (Hsp40) chaperone family in maize (Li, Chen, et al., 2024) and an Arabidopsis mutant defective in embryo development and chloroplast biogenesis (Huang et al., 2009).

Localisation predictions are increasingly complemented by experimental imaging and proteomics. Fluorescent protein fusions (e.g. GFP, YFP, mCherry) expressed via transient or stable transformation allow localisation to be visualised *in planta*. When combined with advanced microscopy techniques such as fluorescence recovery after photobleaching (FRAP), fluorescence loss in photobleaching (FLIP) or Förster resonance energy transfer (FRET), these systems can quantify localisation dynamics and spatial proximity between proteins (Axelrod et al., 1976; Bücherl et al., 2014; Ellenberg et al., 1997; Mullineaux, 2004). Complementary biochemical approaches, including density‐gradient fractionation coupled with western blotting or LC–MS/MS, distinguish organelle‐specific proteomes and validate predicted targeting. Spatial proteomics methods using proximity labelling enable mapping of compartment‐specific interactomes. Combining computation with such experimental datasets allows iterative refinement of computational models, improving predictive accuracy and revealing transient or condition‐dependent localisation patterns in plant cells.

### Future function, localisation and modification directions

While nonspecific annotation methods can be powerful in plant research, developing dedicated tools tailored to plant evolutionary history, polyploidy and genome complexity could substantially increase annotation accuracy and biological relevance. As plant‐specific PTM predictors are developed, computational predictions will continue to guide experimental discovery of post‐translational regulation across plant proteomes. As localisation data for more plant proteomes become available for use in model training, this will improve prediction accuracy for a wider range of model and non‐model plant species. Additionally, expanding tools to cover a wider array of cellular compartments will improve accuracy, decrease uncertainty and increase the applicability of computational predictions for plant biologists.

Connecting protein‐level properties to the cellular pathways and physiological outcomes they influence remains a central goal in plant biology. Integrating functional annotation, PTM prediction and localisation analyses provides a multidimensional view of protein function and could enable prediction of complete cellular pathways. Such hybrid approaches suggest what a protein does and where it acts, as well as how its activity could be dynamically tuned through chemical modification and environmental response. Such integrative annotation is particularly powerful in plant systems, where genome expansion, endosymbiotic compartmentalisation and environmental plasticity intersect. As models increasingly incorporate plant‐specific features such as organelle‐targeting signals and relocalisation, their capacity to deepen our understanding and uncover novel processes will continue to expand.

## PROTEIN STRUCTURE PREDICTIONS

Protein structures enable prediction of catalytic residues, interaction surfaces and binding pockets, allowing for rationalisation of mutation effects and generation of testable hypotheses (Yang et al., 2015; Jumper et al., 2021). In plant biology, where most proteins remain structurally uncharacterised, structure prediction is a powerful tool to explore protein function, evolution and interaction networks (Shahid et al., 2025).

The earliest modern structural prediction method is template‐based modelling, consisting of homology modelling and threading approaches (for a comprehensive timeline of template‐based methods, see Malhotra et al., 2025). Such methods are valuable when there is an appropriate template, which is an experimentally‐determined structure of a protein that exhibits at least 30% amino acid identity (Gromiha et al., 2018). Commonly used template‐based approaches include HHpred (Zimmermann et al., 2018) and SWISS‐MODEL (Waterhouse et al., 2018). These approaches offer transparent alignment control and excel at modelling ligand‐bound states, side‐chains and loops (especially near cofactors). Recent innovations, including I‐TASSER‐MTD (Zhou et al., 2022) and Foldseek (van Kempen et al., 2024) have enhanced the speed and sensitivity of template searches, and improved template integration.

Deep‐learning methods have revolutionised structural biology, generating protein models with increasingly experimental‐level accuracy without requiring a template (Baek et al., 2021; Jumper et al., 2021; Lin et al., 2023). AlphaFold2 (Jumper et al., 2021) sets the standard for single‐chain predictions, while AlphaFold3 and AlphaFold‐Multimer enhance accuracy for protein complexes, ligands, ions and nucleic acid interactions (Abramson et al., 2024; Evans et al., 2022). Alternative tools such as RoseTTAFold (Baek et al., 2021), ESMFold (Lin et al., 2023) and OmegaFold (Wu et al., 2022) provide trade‐offs between accuracy, computational cost and speed. Such methods remain limited when modelling intrinsically disordered regions, large membrane‐integrated assemblies, conformational dynamics and atomic‐level interactions (Akdel et al., 2022; Binbay et al., 2023; Malhotra et al., 2025). Thus, template‐based methods remain important as they can still outperform Artificial intelligence (AI)‐based methods in certain cases, are less computationally intensive and can often produce structures with similar identities and accuracies (Binbay et al., 2023; Malhotra et al., 2025). Importantly, template‐ and deep learning‐based methods can be complementary, with hybrid pipelines (e.g. Phyre2.2, D‐I‐TASSER) improving accuracy for protein complexes and docking (Powell et al., 2025; Zheng et al., 2025).

Plant systems pose unique challenges for structure prediction. Organelle‐targeting peptides complicate modelling, as mature proteins can differ substantially from their genomic encoding (von Heijne, 2002). Furthermore, standard workflows tend not to account for the unique pH, redox state and ionic conditions in which chloroplast and vacuolar proteins fold (Figure 1; Trinh & Masuda, 2022), nor the reliance of many plant proteins on cofactors, metals or pigments (Chigumba et al., 2022; Kroh & Pilon, 2020). Moreover, intrinsically disordered regions, long repeats and coiled‐coil motifs, which are abundant in signalling and defence proteins, can lead to low‐confidence predictions in functionally important regions, such as short linear motifs or phase‐separation drivers (Abramson et al., 2024; Pratt et al., 2025; Ruff & Pappu, 2021).

Recent innovations have made it increasingly feasible to model dynamic assemblies, organelle‐specific environments and complex plant proteomes with realistic accuracy. For example, AlphaFold3 (Abramson et al., 2024) extends modelling to ligands, ions and nucleic acids, while ESMFold (Lin et al., 2023) and OmegaFold (Wu et al., 2022) enable proteome‐level screening through rapid, alignment‐free predictions. Iterative refinement with experimental density maps enhances prediction accuracy, and integrated pipelines, such as MoDAFold, combine prediction with MD to capture conformational variability (Zheng et al., 2024).

Structural predictors are widely used in plant biology and have been used to identify pathogen‐recognition domains in tomato viral resistance proteins (van Grinsven et al., 2022), classify Arabidopsis aspartate proteases (Duan et al., 2023), and identify unique hydrogen bonding profiles in stress‐induced Arabidopsis peroxidases that may interfere with substrate binding (New et al., 2023). Starch metabolism and photosystem stabilising enzymes have also been modelled to generate residue‐level hypotheses, which were subsequently tested by mutagenesis and interaction assays (Berndsen et al., 2025; Ren et al., 2025). AlphaFold‐predicted proteomes for Arabidopsis, maize, soybean and rice, are available in the AlphaFold Protein Structure Database, which provides extensive annotated structures, enabling large‐scale comparative and functional analyses (Fleming et al., 2025).

In experimental structural biology, resolution is defined as the smallest distinguishable feature in the electron density (or potential) map, measured in Ångströms (Å). Though predicted structures cannot be assigned an Å resolution, their accuracy can be evaluated through confidence metrics and by benchmarking against experimental data (Akdel et al., 2022). In AlphaFold2 and AlphaFold3, the predicted local distance difference test (pLDDT) score distinguishes well‐defined regions from flexible regions, while the predicted aligned error (PAE) provides positional uncertainty between residues (Magana Gomez & Kovalevskiy, accessed 5 October 2025). For complexes, the predicted template modelling (pTM) and interface predicted template modelling (ipTM) scores measure overall fold and inter‐chain placement. Regions with high pLDDT are of high confidence in predicted backbone and even side chain conformation. However, pLDDT is not a measure of Ångström resolution and should not be directly correlated with experimental resolution. Definitions and guidance for interpreting these metrics are provided in Table 1.

**Table 1:** Definitions and interpretation of metrics used in this manuscript

Metric	Definition	Interpretation
Resolution (Å)	The smallest distance between two features (e.g. two atoms in the structure) at which they can be differentiated from each other in a protein structure.	Lower numerical values indicate higher resolution structures.
pLDDT	Predicted local distance difference test. The local, per‐residue confidence (0–100) for an Alphafold structure.	Higher scores typically indicate well‐defined regions. Lower scores often indicate flexible or intrinsically disordered regions.
PAE	Predicted aligned error. A global, between residue confidence score (0–30) for Alphafold structures that describes confidence in domain placement and residue packing.	Lower scores indicate greater confidence in the residue positioning in the predicted structure.
pTM	Predicted template modelling. A confidence score for Alphafold predicted protein complex structures.	Scores <0.5 suggest the predicted complex structure is inaccurate.
ipTM	Interface predicted template modelling. An uncertainty score for subunit positioning in Alphafolded protein complexes.	Scores >0.8 indicate high‐confidence predictions. Scores <0.6 indicate failed protein complex structure predictions.
RMSD	Root‐mean‐squared‐deviation. The divergence or motion of the protein backbone from a reference structure.	Higher RMSD values indicate greater flexibility.
RMSF	Root‐mean‐square‐fluctuation. The deviation of an amino acid residue's position from a reference structure.	Higher RMSF values suggest the region in the protein is more flexible/less stable.
Tm (°C)	Melting temperature. The temperature at which 50% of the protein population is folded and 50% is unfolded.	Lower T_m_ values indicates that a protein has a lower thermal resistance, one component of thermostability.
ΔG (kJ/mol)	Free energy. The change in free energy between two protein states, for example, folded & unfolded or bound & unbound.	More negative ΔG values means that the process is more favourable/spontaneous.
ΔCp (J/K)	Heat capacity. The change in heat of the protein as the temperature changes, often measured under constant pressure.	Larger ΔCp values generally indicate a bigger change in solvent exposure/hydrophobic surface area during temperature change.
ΔΔG (kJ/mol)	The change in free energy of the protein upon introduction of substitution(s) to the protein.	Positive ΔΔG often^a^ mean that the substitutions destabilise the measured process (e.g. folding or binding).

^a^ Sign conventions vary across programs because stability changes are not always calculated in the same direction; accordingly, some methods report positive values as destabilising, whereas others report negative values as destabilising. The sign convention for the specific program or method used should therefore be checked before interpretation.

Computational structure predictors have improved in recent years, for example with AlphaFold‐predicted structures achieving sub‐Å Cα root‐mean‐squared‐deviations (RMSD; Table 1) and approaching resolutions commonly seen with experimental structures (Jumper et al., 2021). However, any hypotheses generated from structures or models require experimental validation (for an excellent overview, see: Terwilliger et al., 2024). For example, the following prediction‐based hypotheses have been experimentally validated: residues from predicted structures predicted to be essential for the function of an Arabidopsis phosphate transporter (Liao et al., 2019), anticipated activity of a plant sterol‐biosynthesis enzyme (Rahier et al., 2009) and the expected substrate specificity of an Arabidopsis chloroplast uracil transporter (Witz et al., 2014).

Structural predictions accelerate discovery by enabling rapid structural assessment of protein families and variants, guiding identification of catalytic residues, binding sites and protein–protein interfaces, and providing initial models for fitting low‐resolution cryogenic electron microscopy (cryo‐EM) or crystallography data. While structural prediction tools are already well implemented in plant biology, recent methodological improvements that incorporate dynamics, cofactors and plant‐specific contexts provide additional scope for probing fundamental questions in plant biology and engineering proteins to enhance crop resilience and productivity.

## MODELLING PROTEIN INTERACTIONS

### Protein–protein interactions

Protein–protein interactions (PPIs) underlie nearly every plant cellular process, including signalling cascades, transcriptional regulation, complex assembly and stress response (see review: Cuadrado & Van Damme, 2024). From the regulation of photosystem repair to the transient kinase‐substrate contacts that rewire plant metabolisms under stress, understanding PPIs can provide mechanistic insights into pathways.

Sequence co‐variation and molecular docking approaches can predict how proteins interact in three‐dimensional space. Co‐evolutionary algorithms such as plmDCA (Ekeberg et al., 2014) infer residue‐residue contacts from multiple sequence alignments and can guide docking and identify interaction interfaces when structural data is unavailable. Molecular docking programs, such as HADDOCK (Honorato et al., 2024) and RosettaDock (Lyskov & Gray, 2008), remain standards for modelling biomolecular interactions. They integrate shape complementarity, protein electrostatics and empirical free‐energy scoring to identify low‐energy models. Ensemble‐based or multi‐body docking tools like HADDOCK 2.4 (Honorato et al., 2024) are especially helpful for large scale or flexible systems as they account for conformational heterogeneity and incorporate experimental restraints from cryo‐EM, nuclear magnetic resonance (NMR) or MS cross‐linking.

PPIP (Ding & Kihara, 2019) is a plant‐specific ML approach that integrates sequence, co‐expression and phylogenetic profiles to predict PPIs and has been used with Arabidopsis, maize and soybean proteomes. Additional plant‐specific ML approaches such as ESMAraPPI (Zhou, Lei, et al., 2023) have achieved accuracies of approximately 95%. PlantPathoPPI (Murmu et al., 2025) and MFGAC‐PPI (Wang, Li, et al., 2024) extend this approach to study plant‐pathogen systems, integrating protein language models or neural networks to improve prediction accuracy.

Integrating evolutionary coupling data into structural models has markedly improved the accuracy and efficiency of predicted PPIs. Two recent advancements include RoseTTAFold2‐PPI (Zhang, Humphreys, et al., 2025), a human‐trained deep learning‐based model that integrates co‐evolutionary information into structural model prediction, and ZEPPI (Zhao et al., 2024), a species‐agnostic method that uses co‐evolutionary information from multiple sequence alignments to score predicted PPI models. Emerging AlphaFold‐based methods such as the python package AlphaPullDown2 (Molodenskiy et al., 2025) can be used to streamline high‐throughput proteome‐wide PPI screening with AlphaFold‐Multimer (Evans et al., 2022).

Plant‐specific PPI predictors, such as MFGAC‐PPI, have been used to identify new effector‐receptor and kinase‐substrate pairs relevant to immunity (Wang, Li, et al., 2024). Docking has been used to model plant protein interactions; for example, HADDOCK has been used to model transcription factor and abscisic acid receptor complexes in *Eleusine coracana* (Rani et al., 2024), providing insights into abscisic acid signalling pathways. Similarly, ESMAraPPI has been used to model BIN2–SOS2 interactions in Arabidopsis, clarifying components of the salt overly sensitive (SOS) signalling pathway (Zhou, Lei, et al., 2023).

Experimental methods such as co‐immunoprecipitation, FRET, yeast two‐hybrid, Surface Plasmon Resonance (SPR; Schuck, 1997), cryo‐EM and X‐ray crystallography provide gold‐standard validation of PPIs, but are time‐consuming, limited to high‐abundance or stable complexes, and often impractical for non‐model species. Computational methods can survey thousands of candidate interactions and predict the existence and geometry of binding interfaces. These predictions are increasingly used to guide experimental design to prioritise high‐confidence candidates for testing, identify interface residues for mutagenesis or refine cryo‐EM and crystallographic models.

### Protein–nucleic acid interactions

Transcription, replication, recombination and genome maintenance depend on precise protein–nucleic acid interactions. In plants, these interactions are central to environmental response: transcription factors (TFs) reprogram gene expression under stress and RNA‐binding proteins modulate splicing, stability and translation (Hudson & Ortlund, 2014). Computational modelling accelerates discovery by efficiently predicting the location, strength and conformational outcomes of nucleic acids binding to proteins.

Two complementary modelling approaches are used to predict protein–nucleic acid interactions: structure‐based and sequence‐based approaches. Structure‐based methods use 3D protein and nucleic acid models to predict complex geometry, energetics and specificity by evaluating shape, charge and hydrogen‐bonding patterns at the interface. General‐purpose structure‐based molecular docking tools such as HDOCK (Yan et al., 2020) and NPDock (Tuszynska et al., 2015) have proven effective at locating DNA‐ and RNA‐binding sites. Deep‐learning structure predictors such as RoseTTAFoldNA (Baek et al., 2024) and AlphaFold3 (Abramson et al., 2024) can directly co‐model proteins bound to DNA or RNA, enabling inference of residue‐nucleotide contacts.

Sequence‐based approaches infer protein–nucleic acid binding without requiring structures and include both protein‐centric and genome‐centric strategies. Protein‐centric methods such as DeepDISOBind (Zhang, Zhao, et al., 2022) and iDRNA‐ITF (Wang et al., 2022) classify DNA‐ or RNA‐binding residues using ML models trained on sequence‐derived physicochemical features, including disorder, charge and evolutionary conservation. Genome‐centric methods predict TF binding sites within genomic sequences by integrating motif information, deep learning and DNA shape descriptors, as exemplified by PlantBind, an Arabidopsis‐specific prediction method with some transferability to maize (Yan et al., 2022). Multi‐species plant tools like SeqConv (Shen et al., 2021) and TSPTFBS (Liu et al., 2021) learn TF‐specific sequence determinants across lineages. A recent advance, PTFSpot (Gupta et al., 2024), utilises nucleic acid sequences and AlphaFold‐derived protein structure to identify binding sites and is designed to account for the high natural variability of plant TFs and binding sites, thereby improving generalisability and accuracy across plant genomes.

While plant‐specific protein–nucleic acid interaction predictors have been developed, few studies have applied these tools. One rare example is that Plant‐DTI, a protein‐centred sequence‐based ML approach, was used to successfully predict binding of the transcription factor MeERF72 to the MeSUS1 promoter in cassava, linking stress signalling to sucrose metabolism (Ruengsrichaiya et al., 2022). This lack of examples of applying protein–nucleic acid interaction prediction tools in plant biology is striking. This gap represents a clear opportunity. Predicting interactions between protein and nucleic acids could help establish candidate regulators and target sequences involved in plastid gene expression and plastid‐nucleus communication. Modelling promoter‐protein interactions could guide promoter engineering or promoter edits to tune tissue‐ or stress‐inducible expression. Computationally predicting interactions with known stress response genes and proteins could allow triage of transcription factors and cis‐elements underlying abiotic‐stress responses before committing to time‐consuming genetic manipulation. As these computational predictions generate specific, testable binding hypotheses, they naturally pair with targeted experimental validation strategies.

Once identified, predicted TF binding proteins can be experimentally validated via affinity assays, for example by electrophoretic mobility shift assays (EMSA), pull‐down assays or filter‐binding assays (Guille & Kneale, 1997; Hall & Kranz, 1999; Hellman & Fried, 2007; Welsh & Cantor, 1984). Function can be assessed using reporter assays or CRISPR base editing *in planta*. Predicted genomic binding positions can also be validated with ChIP‐seq peak positions or high‐resolution methods such as ChIP‐exo (Park, 2009; Rhee & Pugh, 2011). For RNA‐centric predictions, contact sites can be experimentally validated through eCLIP/CLIP‐seq (Stoute & Liu, 2021; Van Nostrand et al., 2016) and mutational profiling. Iterative cycling between prediction and experimental validation could improve confidence for single interactions and yield better trained models over time.

### Protein‐metabolite and protein‐ligand interactions

Proteins and metabolites form the chemical fabric of plant life. Every step of primary metabolism, hormone perception and stress signalling depends on protein‐small‐molecule interactions (for a review, see: Fischer et al., 2025). Protein‐metabolite interactions (PMIs) are especially important in plants because their metabolic networks are expansive, branched and environmentally responsive (for a review, see: Rao & Liu, 2025).

Predicting how metabolites bind enzymes can provide insights into allosteric control and substrate channelling and guide mutational strategies to redesign flux. Similarly, modelling interactions between proteins and auxin, abscisic acid, gibberellins and brassinosteroids clarifies how hormones (and analogues) can modulate signalling pathways. Beyond signalling, metabolite docking can be used to identify enzyme‐substrate relationships in pathways such as amino acid metabolism or metal‐ion coordination in catalytic centres.

Methods for modelling PMIs typically combine geometric analysis, biophysical complementarity and docking‐based free energy estimation to predict metabolite‐binding sites. Structure‐based PMI predictors require a protein structure and search for surface cavities or pockets compatible with small‐molecule ligand shapes and electrostatics. For example, Patch‐Surfer2.0 (Zhu et al., 2015) describes protein surfaces as collections of 3D patches defined by curvature, hydrophobicity and charge distribution. While structure‐based predictors identify pockets *de novo* from the 3D protein structure, template‐based methods, such as ConCavity (Capra et al., 2009), integrate sequence conservation and structure‐based methods to map binding sites onto an input protein. Recently, pipelines such as CB‐Dock (Liu et al., 2020) have been developed that automate cavity‐based binding pocket identification and subsequent molecular docking with AutoDock Vina, a commonly used program for protein‐ligand docking (Eberhardt et al., 2021).

Most recent innovations for predicting PMIs involve developing ML and deep‐learning frameworks, and these methods now dominate the field. Kalasanty (Stepniewska‐Dziubinska et al., 2020) and DeepPocket (Aggarwal et al., 2022) use 3D convolutional neural networks to identify protein–ligand interaction pockets. Recent AutoDock Vina derivatives have begun to leverage hardware acceleration (e.g. Vina‐GPU 2.1, Tang et al., 2024) for orders‐of‐magnitude faster screening, and to incorporate convolutional neural networks for scoring for improved accuracy (e.g. GNINA1.0, McNutt et al., 2021). Databases like DrugBank 6.0 (Knox et al., 2024), ChEMBL (Zdrazil et al., 2024) and BRENDA (Chang et al., 2021) provide training and benchmarking data for PMI models. For plant‐specific models, expanding plant‐specific databases can supply metabolic context (e.g. AraCyc, Hawkins et al., 2025). Integrating these databases with ML allows plant scientists to predict new enzyme‐metabolite or receptor‐hormone pairs directly from sequence and pathway annotations.

PMI modelling has deepened our understanding of phytohormone signalling and metabolism. For example, docking and MD simulations have been used to examine the binding of abscisic acid to PYR/PYL/RCAR receptors, revealing signal activation‐associated conformational changes (Liu et al., 2023; Niranjan et al., 2021). Similar workflows have screened phytochemical agonists of abscisic acid receptors (Shanthappa et al., 2025), and docking of auxin and synthetic auxin herbicides to TIR1/AFB‐Aux/IAA complexes has clarified how minor modifications alter degradation dynamics in *Sisymbrium orientale* (de Figueiredo et al., 2022). These studies demonstrate how phytohormone binding prediction can guide fundamental understanding and rational agrochemical design. Recently, the development of HNCGAT, a plant‐specific neural‐network framework, has extended PMI prediction to entire Arabidopsis proteomes and highlighted previously unrecognised links between guanine and carboxylesterases (Zhou et al., 2024).

Predicted PMIs can be experimentally validated with a growing suite of proteome‐wide methods. Thermal proteome profiling (Molina et al., 2013) detects ligand binding via shifts in melting temperature; LiP‐small‐molecule mapping (Piazza et al., 2018) identifies PMIs from modifications in proteolysis upon ligand binding; and PROMIS (Veyel et al., 2018), developed for Arabidopsis, couples size‐exclusion chromatography with proteomics and metabolomics to identify PMIs from co‐elution patterns. More recently, MIDAS (Hicks et al., 2023) integrates MS with equilibrium dialysis to detect weak and transient allosteric interactions. Iterating predictions with experimental data produces quantitative system‐wide and mechanistically grounded maps of metabolic regulation.

### Future protein interaction modelling directions

Plant‐specific challenges shape both PMI modelling and output interpretation. Expanded TF families with subtle motif preferences, frequent paralogy and lineage‐specific cis‐regulatory grammar all limit the utility of animal‐trained models for plant questions (Zenker et al., 2025). Organelle‐targeted DNA/RNA‐binding proteins introduce additional complications, as well as chloroplast and mitochondrial nucleic acids which differ in packaging and composition (Nishimura, 2024). These contexts illuminate the importance of incorporating organelle‐localisation predictions into plant protein interaction predictors and training models on species‐aware data when possible.

While existing methods have been used to study plant proteins and pathways, protein interaction predictions remain an underutilised method with high potential for increased adoption in plant biology over the coming years. As more plant‐specific models are developed and more experimentally‐verified plant interactions become known, iterative training and benchmarking could close the gap between predictive and experimental interactomics, enabling *in silico* reconstruction and rational rewiring of dynamic signalling and metabolic networks across plant species.

## PROTEIN FLEXIBILITY AND STABILITY

Protein function is inherently linked to flexibility and stability. Enzymes alter their conformations as they assemble into complexes, bind cofactors and substrates, and respond to cellular cues such as temperature, pH or stress‐induced signalling cascades. Throughout these processes, enzymes must maintain sufficient flexibility to perform their functions and sufficient stability to prevent denaturing. Stability predictors and conformational dynamics simulations link plant protein sequence and structure to performance under different conditions.

### Stability predictions

Protein stability reflects how well a protein maintains its folded structure under changing conditions. It represents a combination of both thermostability, typically measured by the melting temperature (T_m_), and thermodynamic stability, defined by parameters such as the unfolding free energy (ΔG), enthalpy, entropy and heat capacity (ΔC_p_) (Table 1; Pucci & Rooman, 2014; Alfano et al., 2017; Miotto et al., 2019). Protein stability is important because mutations or environmental fluctuations can alter folding and, consequently, function. Furthermore, there is often a trade‐off between function and stability by which mutations that enhance protein activity tend to be destabilising, necessitating compensatory mutations to maintain stability (DePristo et al., 2005; Tokuriki & Tawfik, 2009). Computationally predicting stability allows rapid *in silico* screening of mutation sets before experimental testing (Figure 2). These predictions are valuable because stability affects solubility, protein yield and the range of biophysical methods suitable for characterisation.

Current methods for stability prediction include structure‐based energy approaches, elastic‐network and normal‐mode frameworks, and graph‐ and network‐based tools. Structure‐based energy functions predict the change in folding or binding free energy (ΔΔG) caused by amino acid substitutions (Table 1). Common structure‐based stability approaches include FoldX (Delgado et al., 2019), Rosetta ddG_monomer (Kellogg et al., 2011), cartesian_ddG (Frenz et al., 2020) and flex ddG for interfaces (Barlow et al., 2018). These models rely on empirical or physics‐based energy functions, sometimes referred to as ‘effective force fields’, to quantify how specific mutations stabilise or destabilise a protein or interface. Effective force fields are energy functions that estimate free energy changes based on factors such as van der Waals interactions, hydrogen bonding, solvation and electrostatics, that are estimated based on the protein model. It is important to note that effective force fields are different to the classical molecular mechanics force fields used in MD simulations, which are discussed in the next section.

Elastic‐network and normal‐mode frameworks, such as DynaMut2 (Rodrigues et al., 2021), complement structure‐based energy approaches by estimating how substitutions alter flexibility and vibrational entropy, providing insights into thermal sensitivity, conformational dynamics and allosteric communication.

Finally, graph‐ and network‐based rigidity analyses treat a protein structure as a network of interactions. Such methods can predict complementary stability effects of mutations: CNAnalysis (Krüger et al., 2013; Pfleger et al., 2013) identifies rigid clusters and can highlight ‘weak spots’ contributing to thermal instability, while WebPSN (Felline et al., 2022) constructs network or pathway maps and can predict allosteric hotspots, residues likely to be involved in long‐range interactions or communication. These data can guide protein engineering, enzyme optimisation and interpretation of plant protein function under changing environments.

Recent innovations in stability prediction combine advances in physics‐based modelling, ML and large‐scale structural datasets to improve speed and accuracy. Deep‐learning frameworks such as DDGemb (Savojardo et al., 2025) use neural networks trained on experimentally measured stability changes to predict the thermodynamic effects of mutations with high precision. Pretrained deep‐learning methods such as RaSP (Blaabjerg et al., 2023) have also been developed for high‐throughput stability screening of entire proteomes. Some methods now integrate multiple sequence alignment‐based protein language models for increased universality and predictive power (e.g. MSA Transformer, Rao et al., 2021). Reducing biases in predictions is another area of innovation. Recent methods use more balanced training datasets (e.g. PremPS, Chen et al., 2020) or ensemble predictors that combine multiple ML approaches (e.g. MAESTRO, Laimer et al., 2015) to mitigate biases arising from overtraining on experimental destabilising mutation data.

Stability predictors remain underutilised in plant biology, despite recent studies that illustrate their potential to connect molecular evolution, protein engineering and environmental adaptation. DynaMut has been used to explore how mutations affect bryophyte Rubisco stability and flexibility, providing insight into Rubisco's evolutionary history (Anand & Alam, 2025). WebPSN v2.0 has been used to identify specific phytochemicals with dense interaction networks with IL‐2‐inducible T‐cell kinase, a drug target that plays a critical role in T‐cell signalling, suggesting enhanced binding affinity and specificity (Ahmed et al., 2025).

Stability prediction has also guided protein engineering efforts. Using CNAnalysis, Friberg et al. (2025) identified unfolding nuclei and weak structural regions in patatin from *Solanum cardiophyllum*. Experimental validation found no increased thermostability but did identify a variant with improved pH tolerance. Rosetta ddg_monomer has been used to screen mutations in a *Stevia rebaudian*a glycosyltransferase, filtering for predicted stabilising variants prior to validation (Go et al., 2023). Similarly, cartesian ddG has been used to identify stabilising mutations in an enzyme involved in magnolol biosynthesis in *Magnolia officinalis* (Yang et al., 2024).

These tools have also been used to study the mechanisms driving plant adaptation. For example, FoldX predicted that Coenzyme A binding substantially lowered the folding energy of the heat‐adapted Arabidopsis N‐acetyltransferase NATA2 relative to NATA1, accounting for its greater stability (Hameed et al., 2024). FoldX has also been used to identify why soybean, but not Arabidopsis, receptors recognize the bacterial PAMP flg22 from *Ralstonia solanacearum (*Wei et al., 2020).

Stability predictions can be experimentally validated using a range of complementary biophysical methods that quantify stability parameters such as T_m_, unfolding ΔG and ΔC_p_ (for a review, see: Atsavapranee et al., 2021). Such methods include thermal shift assays using dyes to measure global stability by monitoring fluorescence changes as proteins unfold upon heating (Biggar et al., 2012; Joiner & Fromme, 2021; Lavinder et al., 2009). Additional validation approaches include chemical denaturation assays, circular dichroism spectroscopy and differential scanning calorimetry, which provide folding free energies, measurement of secondary‐structure content and loss during unfolding and ΔC_p_ throughout unfolding (Table 1; Pace & Scholtz, 1997; Sato et al., 2000; Greenfield, 2006a; Greenfield, 2006b; Johnson, 2013). Methods such as NMR and hydrogen‐deuterium exchange coupled to MS can also validate predictions by identification of residue‐level differences in solvent exposure and stability (Cieplak‐Rotowska et al., 2018; Dyson & Wright, 2004). At proteome scale, thermal proteome profiling combines temperature‐dependent denaturation with MS to determine which proteins unfold at given temperatures, allowing high‐throughput validation of trends (Jarzab et al., 2020).

### Molecular dynamics simulations

MD simulations provide insights into enzyme flexibility and motion by modelling atomic motion over time, revealing conformational changes, binding pathways and allosteric networks. In practical terms, the primary output of an MD simulation is an atomic trajectory, which is a time‐resolved ‘movie’ of the protein. These trajectories can be used to compute metrics that capture protein dynamics. In MD, atomic trajectories are computed by numerically solving Newton's equations of motion under defined thermodynamic simulation conditions (e.g. constant temperature and sometimes pressure), referred to as a thermodynamic ensemble. These calculations depend on a molecular mechanics force field: a set of mathematical terms and parameters that specify how atoms interact, including how bond lengths and angles can deform and how non‐bonded atoms attract or repel one another.

MD can be studied at different resolutions via all‐atom and coarse‐grained simulations (for review, see: Hollingsworth & Dror, 2018). MD simulation programs, such as GROMACS (Páll et al., 2020) and AMBER (Case et al., 2005; Case et al., 2023), vary in their ease of use and tend to have large user support communities. All‐atom simulations are suitable for detailed mechanistic studies as they represent every atom in the protein, offering the highest‐resolution insights into conformational dynamics, ligand binding, ion coordination and hydration networks (Hollingsworth & Dror, 2018). All‐atom MD simulations are computationally intensive and can be limited to sampling timescales of nanoseconds or a few microseconds (Zwier & Chong, 2010). Enhanced‐sampling methods can augment standard MD by expanding the timescales on which atomic trajectories can be captured, as these methods improve simulation of slow or rare large conformational changes (for review, see: Bernardi et al., 2015; Hénin et al., 2022).

Coarse‐grained simulations simplify proteins by grouping atoms into pseudo‐particles (Hollingsworth & Dror, 2018; Marrink & Tieleman, 2013). This dramatically reduces computational cost, allowing simulations of very large macromolecular assemblies, such as thylakoid membranes, photosystem complexes or organelle‐scale systems over longer timescales (up to milliseconds or longer depending on hardware). The trade‐off is reduced atomic detail and accuracy, especially for side‐chain interactions and for small conformational changes. Coarse‐grained simulations can be run in general MD simulation programs like GROMACS (Páll et al., 2020) and DL_POLY (Smith et al., 2002), using coarse‐grain force fields such as MARTINI (Souza et al., 2021) and UNRES (Sieradzan et al., 2022; Ślusarz et al., 2022; Ślusarz et al., 2025).

Recent innovations have improved the power of MD simulations. In addition to experimentally‐obtained structures, predicted structures are now routinely used as starting structures for MD simulations (Nussinov et al., 2023). These structures are protonated and parameterised with physiologically‐relevant ions, cofactors or redox states and equilibrated under specified solvent or membrane conditions (Figure 1; Nerenberg & Head‐Gordon, 2018; He et al., 2022; Feng et al., 2023). New software, such as GENESIS 2.1 (Jung et al., 2024), has been developed that can run all‐atom, coarse‐grained and quantum mechanics/molecular mechanics simulations, allowing for ease of multi‐scale simulations.

Advances in GPU‐accelerated engines, seen with GROMACS (Páll et al., 2020) and AMBER 22 (Case et al., 2023), and adaptive sampling algorithms (Kleiman et al., 2023) extend simulations toward hundreds of microseconds, bridging simulation and biological timescales. ML‐enhanced MD approaches such as DeepDriveMD (Lee et al., 2019) can identify the most important motions and guide simulations toward new, unexplored protein conformations, while TorchMD‐NET (Pelaez et al., 2024) provides network potentials that can be used within such adaptive frameworks.

Although less frequently applied to plant systems than to animal or microbial models, MD has been used to explore conformational flexibility, stability and binding interactions under biotic and abiotic stresses. For example, MD was used to model the molecular mechanisms of copper ion adsorption to *Glycyrrhiza glabra* roots (Pirsalami et al., 2021). Similarly, docking and MD approaches were used to identify transcriptional regulators that confer salinity tolerance in *Triticum aestivum*, through predicting stable protein‐DNA interactions (Hassan et al., 2022). MD has also been used to study plant‐microbe contexts, protein–ligand interactions and plant‐derived metabolites. GROMACS has been used to model a rice glucanase‐GTP complex to study its role in pathogen defence (Jha et al., 2022). Similar protein‐ligand interactions were simulated to evaluate Asteraceae‐derived thiophenes (secondary plant metabolites) as potential inhibitors of human cathepsin D, a lysosomal protease (Ibrahim et al., 2022). MD‐based binding energy analysis was used to simulate adsorption of compounds found in the naturally anti‐corrosive *Citrus reticulata* leaves to a copper surface to assess potential corrosion inhibition activity from simulated binding energies (Xiang & He, 2021).

While MD provides atomic‐scale trajectories of motion, experimental data serve as the benchmark for testing whether simulated dynamics correspond to real molecular ensembles (for reviews see: Hospital et al., 2015; Hollingsworth & Dror, 2018). Structural and conformational changes predicted by MD can be validated with structural biology approaches including X‐ray crystallography, cryo‐EM and NMR spectroscopy. Hydrogen‐deuterium exchange coupled to MS or NMR can also experimentally validate MD simulations as it quantifies changes in solvent accessibility and backbone flexibility that can be qualitatively compared to root‐mean‐square‐fluctuation (RMSF) profiles. RMSF is a per‐residue ‘wiggle’ metric: it measures how far each residue moves from its average position over the simulation, with higher RMSF indicating more flexible regions and lower RMSF indicating more rigid regions (Table 1; Cieplak‐Rotowska et al., 2018).

Spectroscopic methods also provide complementary validation of structural dynamics. Circular dichroism and fluorescence spectroscopy can monitor temperature‐ or ligand‐induced conformational shifts predicted by MD. Functional and kinetic assays provide an additional layer of validation, particularly for plant enzymes and receptors where simulations predict the effects of specific mutations or ligand interactions. Site‐directed mutagenesis followed by enzyme kinetics, binding assays (e.g. Isothermal titration calorimetry or SPR) or thermal shift analysis can confirm the energetic and mechanistic consequences of motions observed *in silico*. For membrane proteins and signalling complexes, lipid nanodisc (Denisov & Sligar, 2016) or proteoliposome reconstitution (Rigaud & Lévy, 2003) can enable verification of MD‐predicted conformational transitions under near‐physiological conditions.

### Future protein flexibility and stability directions

Taken together, stability predictors and MD simulations provide complementary insights into how plant proteins maintain function while navigating environmental volatility. Predictors efficiently identify stabilising or destabilising substitutions, while MD describes the mechanistic basis by simulating which structural features shift, stiffen and/or remain plastic. Stability predictors and MD simulations remain underutilised in plant biology, and existing methods can be applied to study the flexibility and conformational changes associated with function for diverse plant proteins. Expanding plant‐specific training datasets and benchmarking efforts for stability predictors will be key to improving prediction accuracy and unlocking the full potential of stability modelling for plant biochemistry. It is currently challenging to run simulations over biologically‐relevant timescales to capture large and slow conformational changes that can provide insights into a protein's mechanism of action, and this will be made more easily achievable through continued hardware advancements, such as GPU acceleration.

## PERSPECTIVES

Computational approaches are reshaping how plant biologists study proteins, providing the means to generate and test mechanistic hypotheses before entering the slow and costly realm of *in planta* experimentation (Figure 2). Integrating computational methods that span evolutionary, structural and biophysical scales (Box 1), researchers can now explore questions inaccessible through experimentation alone. Selected case studies cited throughout this review are summarised in Table S2, linking specific computational approaches to the experimental validation used to address plant biology questions.

Box 1 **Main points**

1. *Evolution:* ASR can probe the evolutionary history of plant proteins, identifying key substitutions driving functions and stability.
2. *Function:* Computational annotation predicts protein function, regulation and cellular context.
3. *Structure:* Structure prediction methods provide atomic‐level hypotheses for uncharacterised proteins, offering insight into sequence‐structure–function relationships.
4. *Interaction:* Docking and co‐evolutionary models map protein, nucleic acid and metabolite networks.
5. *Dynamics:* Stability predictors and MD simulations reveal protein flexibility and response to environmental conditions.

### From data to discovery: selecting the right approach

**Figure 3:**
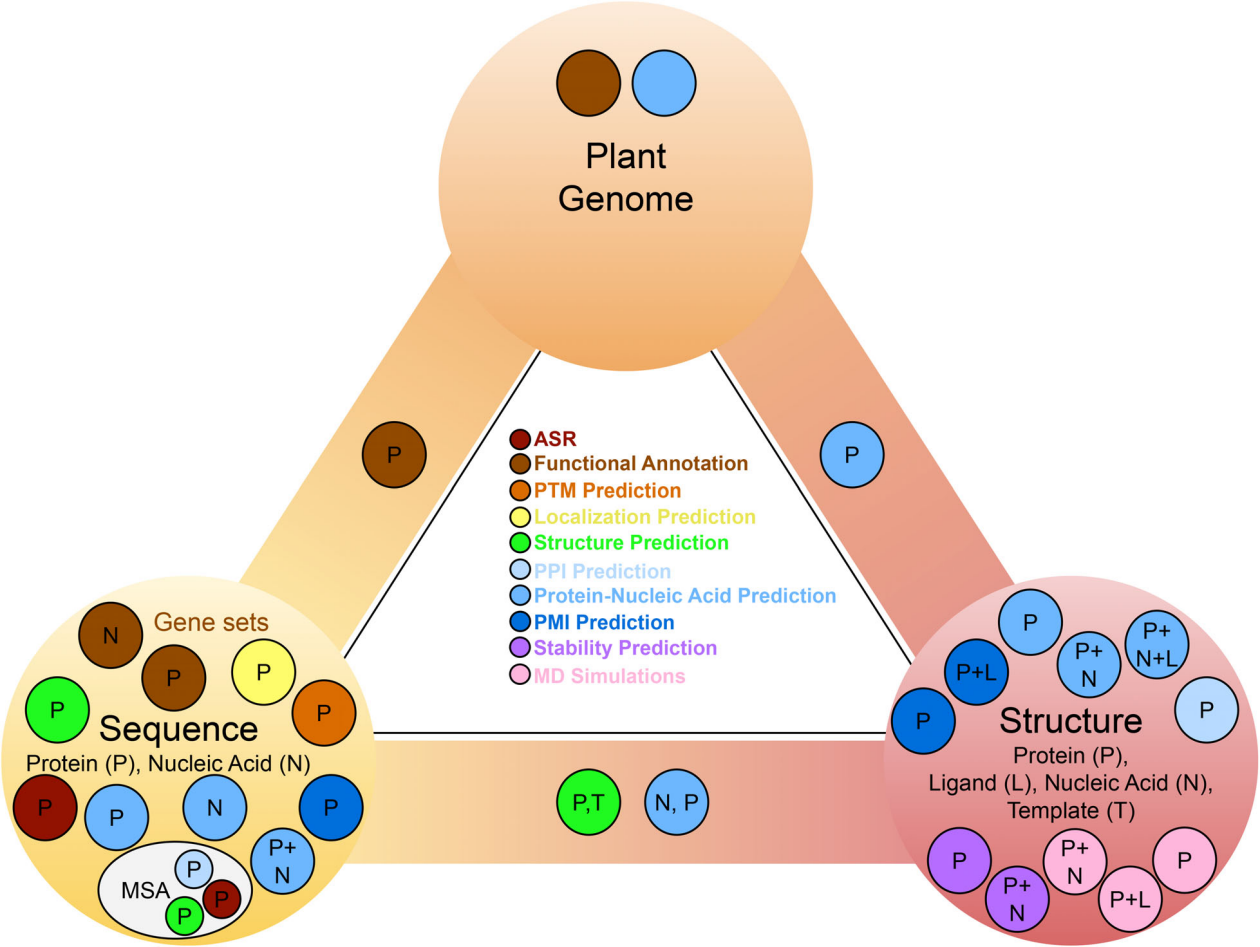
Summary of data inputs required for different computational methods. Different computational approaches require distinct types of input data, which can be broadly grouped into three categories shown at the vertices of the triangle: genomes, sequences and structures. The sides of the triangle represent the integration of both data types at the respective vertices. Coloured circles indicate computational approaches that can be used given the available inputs. If plant genome(s) are available, methods can be used for functional annotation or prediction of protein–nucleic acid interactions by scanning the genome for potential binding sites. If a protein sequence is known, tools can be used for functional annotation, localisation prediction, PTM prediction, structure modelling and predicting interactions between proteins and nucleic acids or metabolites. Specific tools can also be used to reconstruct ancestral sequences given a single input protein sequence. With a multiple sequence alignment (MSA) of related proteins, additional analyses such as PPI prediction become possible. If a nucleic acid sequence is provided, it can be annotated or scanned for common protein‐binding motifs (e.g. transcription factor binding sites). When both protein and nucleic acid sequences are available, protein–nucleic acid interaction predictions can be completed. If protein structures are available, protein stability can be predicted and molecular dynamics simulations can be performed. Furthermore, from a single protein structure, likely protein‐, nucleic acid‐ or metabolite‐binding sites can be identified. When paired with candidate structures of interacting partners (proteins, ligands, metabolites or nucleic acids), interaction modelling, molecular docking and MD simulations can be conducted to assess stability and conformational flexibility of the complex. For a list of specific methods, with inputs, outputs and accessibility, see Table S1. ASR, ancestral sequence reconstruction; L, ligand; MD, molecular dynamics; MSA, multiple sequence alignment; N, nucleic acid; P, protein; PMI, protein‐metabolite interactions; PPI, protein–protein interactions; PTM, post‐translational modification; T, template.

Data can be limited: many plant species lack a reference genome, transcriptomic assemblies can be incomplete and obtaining new sequencing data may be beyond the scope of a given project. As a result, computational strategies must often begin with whichever data are already available. Figure 3 provides a simplified, input‐driven decision tree that highlights which computational approaches are possible given different available data types. From a methods perspective, ASR requires protein sequences and/or multiple sequence alignments (MSAs). Functional annotation can use nucleic acid sequences, plant genomes and/or protein sequences. PTM and localisation predictions use protein sequences. Structure prediction can use protein sequences, MSAs or template structures. Protein stability and interaction modelling can use one or more sequences, plant genome and/or structure as input. A more comprehensive guide to specific programs, required inputs and outputs is included in Table S1.

### Integrating methods into multi‐step discovery pipelines

**Figure 4:**
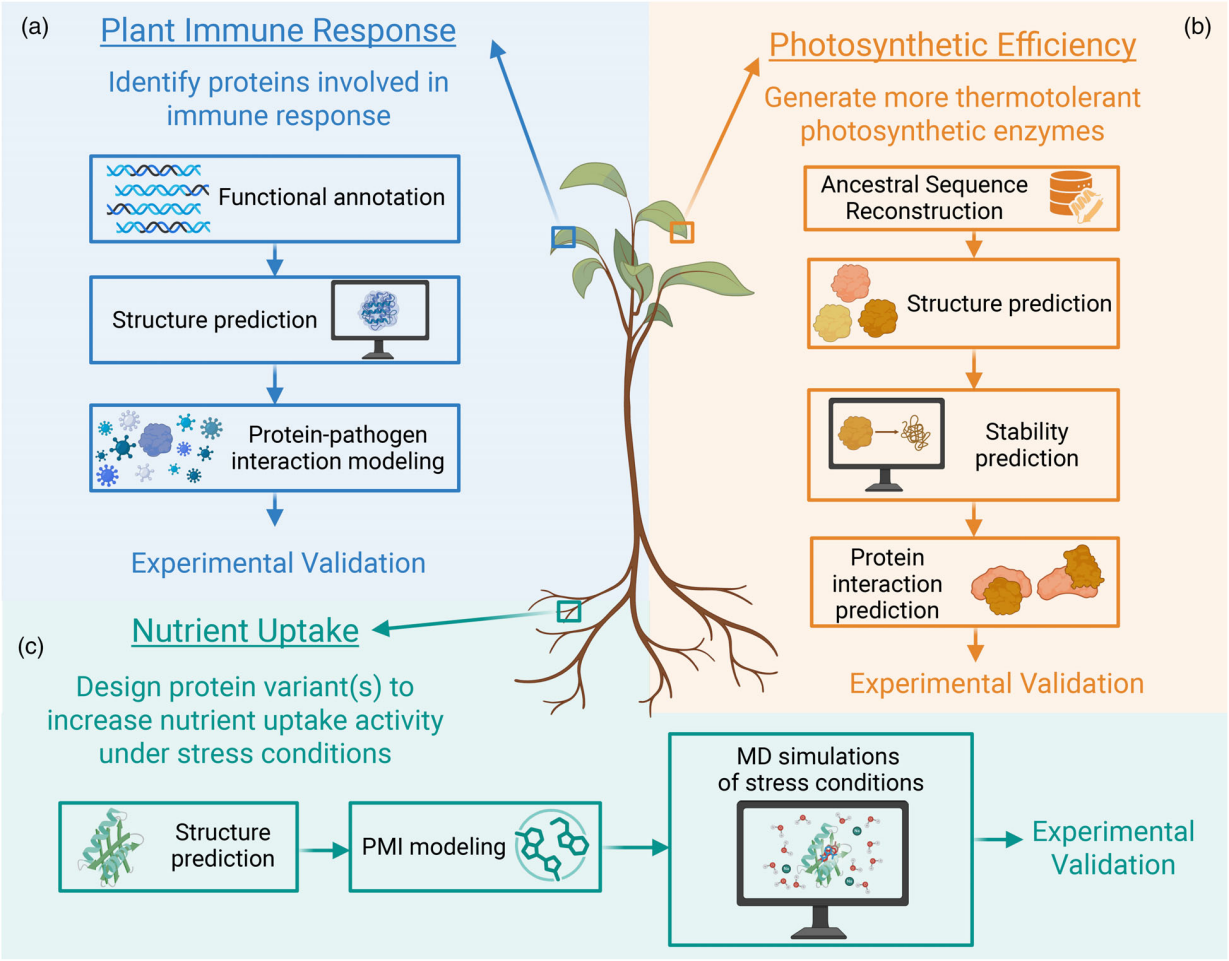
Schematic showing opportunities to use computational approaches to bridge fundamental plant discovery and agricultural application. The wider adoption of computational approaches could be used to address a broad range of plant‐related questions, from basic discovery to translational engineering. Examples include (a) screening plant genomes and proteomes to identify proteins involved in immune response pathways, (b) engineering more thermotolerant photosynthetic enzymes to enhance photosynthetic efficiency under climate stress and (c) designing protein variants that improve nutrient uptake under limiting or stress conditions. Across these applications, combinations of functional annotation, structure prediction, protein interaction modelling, stability prediction and molecular dynamics simulations can be used to generate mechanistic hypotheses and narrow down candidate proteins or compounds for experimental validation. Together, these approaches illustrate how multi‐step computational pipelines can accelerate fundamental plant protein research toward understanding plant protein function and engineering plant proteins for enhanced resilience and productivity. Created in BioRender. Bohling, S. (2026) https://BioRender.com/jwo9a42.

The exploratory power of computational biology can be increased when methods are integrated into multi‐step computational workflows. There is great potential for combining evolutionary, structural and dynamic analyses to study plant proteins, as illustrated by Figure 4. For example, genome‐scale screening could be used to study plant immune response, with possible translation for crop health, by using computational functional annotation approaches to identify candidate immune receptors. Structural and stability modelling could be used to deepen our understanding of enzymatic thermal adaptation by identifying potential thermotolerant variants of photosynthetic enzymes. This has translational potential for engineering crop resilience under elevated temperatures. Docking and MD simulations could be used to guide protein design for enhanced nutrient uptake or stress resilience.

Such integrated pipelines enable efficient filtering of expansive omics datasets to identify potential interactors by sequence conservation, predicted structure and interaction likelihood. By applying experimental validation to only the most promising subset, this approach dramatically reduces experimental workload and cost, a critical advantage in plant systems where each transformation or mutant line can take months to generate (Figure 2).

### From methods to mechanisms

The next frontier for plant computational biology lies in linking molecular predictions to physiological and environmental contexts. ML frameworks trained on plant‐specific omics and structural data will improve accuracy for predicting cofactor‐ and metal‐binding sites and account for organellar context. Integrating computational outputs with genomics, transcriptomics, proteomics and metabolomics will provide a systems‐level view of plant regulation and adaptation. Large‐scale databases that connect protein structure, expression patterns and metabolite profiles are key to this integration. Expanding community resources such as metabolite databases, AraCyc (Hawkins et al., 2025), PhosPhAt (Zulawski et al., 2012) and P^3^DB (Yao et al., 2014) to include predicted structures and interactions could enable mapping of entire plant biochemical networks with unprecedented depth.

### Linking computation to experimental validation

Computational predictions reach their full potential when coupled with experimental and systems‐level data that validate predictions, grounding them in a physiological context. As *in planta* work remains time‐intensive, iterative pipelines combining *in planta*, *in silico* and semi‐synthetic methods will be critical for making predictive biology routine in plant research. Semi‐synthetic approaches, such as heterologous expression in *E. coli* or yeast, offer rapid, low‐cost systems for testing plant protein activity, ligand binding or thermostability by acting as an intermediary between *in silico* models and *in planta* systems. Plant‐derived systems such as tobacco BY‐2 suspension cells (Nagata et al., 1992) or wheat‐germ extract (Harbers, 2014; Roberts & Paterson, 1973) provide more plant‐like expression environments, while cell‐free and microfluidic screening technologies (Romero et al., 2015; Silverman et al., 2020) can further increase throughput. Remaining challenges for building efficient, transparent and reproducible validation frameworks are summarised in Box 2.

Box 2 **Remaining questions**

1. *Plant‐trained models:* How can we expand plant‐specific training data, including negative data, to improve model calibration and reduce cross‐kingdom bias?
2. *Environment‐aware prediction:* Which parameters best approximate chloroplast, mitochondrion, vacuole and apoplast environments (pH, redox, crowding, ionic strength and composition), and how should these be incorporated into structure, dynamics and docking pipelines?
3. *Integrative validation:* What are efficient pipelines to integrate computational predictions, *in silico* filtering, heterologous assays and *in planta* validation?
4. *Transparency and reproducibility:* What benchmarks and reporting standards will help yield reproducible, comparable results across plant lineages and clearly communicate uncertainty?
5. *Plant biology adoption and training:* Which tutorials, notebooks and turnkey workflows can be developed to best accelerate adoption by plant biologists with limited computational background?

### The need for plant‐trained models

Plant biology presents unique computational challenges. Polyploidy, gene duplication and frequent hybridisation events complicate ortholog inference and co‐evolutionary analyses (Guo et al., 2023). The folding and interaction dynamics of plant proteins depend on the subcellular compartment in which they operate, since chloroplasts, mitochondria and peroxisomes each maintain distinct ionic and redox environments (Figure 1). Furthermore, stress responses, hormone signalling and secondary metabolism add regulatory layers and feedback loops that can be difficult to capture in generic models (Aerts et al., 2021; Bittner et al., 2022; Li, Jiang, et al., 2024).

To address these challenges, next‐generation predictive frameworks must become plant‐trained and environment‐aware. Plant‐trained models should incorporate species‐specific sequence features, organellar targeting peptides and evolutionary patterns, while also accounting for negative examples to calibrate predictions and reduce cross‐kingdom bias. Environment‐aware modelling, in turn, should approximate the physicochemical conditions of the relevant subcellular compartment, enabling predictions that better reflect *in planta* function.

### Increasing adoption of computational methods in plant biology

While many computational methods have existed for decades, recent advances have greatly improved their usability. The growing number of available web servers and simplified interfaces are lowering the barrier to adoption by researchers without formal computational training. The practical barrier to adoption is often computational, not conceptual and while many methods are accessible through web servers (minimal local resources), others benefit from local compute or high‐performance computing (HPC). Local compute refers to running software on a lab workstation (or a small lab server), whereas HPC typically refers to shared university, national or consortium clusters that schedule jobs across many CPUs and/or GPUs (specialised processors often used to accelerate deep learning). In Table S1, ‘Accessibility’ indicates whether a tool is available as a web server (no installation), source code/software package (local installation; e.g. ‘R package’) or a packaged/cloud‐style distribution (e.g. Colab notebooks, Docker images or preconfigured machine images).

As a general guide, sequence‐based annotation and many predictors run comfortably on a standard laptop or desktop. In contrast, structure prediction at scale, local deep‐learning workflows and long all‐atom MD simulations often require GPU acceleration and/or HPC scheduling. Cloud platforms can lower the barrier by providing on‐demand GPUs for bursty workloads (e.g. structure prediction or deep‐learning inference) and scalable CPU resources for large‐scale screening tasks, but they also introduce costs and data‐management considerations. Table S1 provides further information on which discussed methods can be used on a standard laptop, a lab workstation or access to HPC.

While resource barriers may exist for running large‐scale or computationally heavy jobs, many existing tools have freely available web servers that can efficiently run small‐scale jobs and thus can be widely adopted by plant biologists regardless of their current lab computational infrastructure. When considering implementing computational methods to study a biological question, we encourage matching tool choice to available resources, starting with web‐based pipelines where possible and escalating to local/HPC workflows only when the biological question requires it. Additionally, for labs with limited computational infrastructure, collaboration with computational groups or institutional research‐computing teams can provide an efficient entry point to GPU‐ and HPC‐enabled workflows. Many universities and regional/national consortia also offer shared computing allocations and user support (often via short applications or internal request processes), lowering the barrier to running resource‐intensive analyses. Tips for getting started using computational tools to answer plant biology questions can be found in Box 3.

Box 3 **Actionable tips for getting started applying computational methods to plant biology questions**

1. *Define Goals:* Establish the biological question and the output(s) required to address the question (e.g. prioritise mutations, predict localisation, rank variant stability).
2. *Select Tool(s):* Determine applicable methods depending on available inputs, method outputs and available computational resources; start with a web server version of the required computational tool, moving to local/HPC if needed.
3. *Test & Validate:* Run a pilot (i.e. run on a small subset of inputs) before scaling up and plan at least one experiment to test a subset of computational outputs (e.g. assay, microscopy, mass spectrometry).
4. *Record Parameters:* Keep clear documentation for each computational run, including all inputs, tool and version, default and modified settings, date and any run‐specific parameters needed for reproducibility, such as random seed(s), where relevant.
5. *Redundancy & Replicability:* Run jobs in replicates and compare outputs from multiple tools when possible.

The community's next challenge is to ensure FAIR (Findable, Accessible, Interoperable, Reusable) data standards and transparent benchmarks, enabling reproducible results across lineages and laboratories (Wilkinson et al., 2016). Additionally, similar to the health AI model cards proposed by the Coalition for Health AI (CHAI), development of shared plant ‘model cards’ documenting input requirements, confidence metrics and performance benchmarks would promote transparency and cross‐tool comparability (CHAI, 2024; Gilbert et al., 2025).

## CONCLUSIONS

Multi‐step hybrid pipelines that combine AI‐driven structure prediction, molecular simulation and experimental multi‐omics data will deepen our understanding of how plant proteins fold, interact and evolve under changing environments. Ultimately, these advances will allow plant scientists not only to interpret the molecular diversity of plants but also engineer enzymes, regulatory proteins and pathways that enhance resilience, efficiency and sustainability. By integrating plant‐specific data, transparent validation and community training, computational plant biology is poised to move from description to prediction and from prediction to design, empowering a new era of mechanistic and rational plant science.

## Author Contributions

SMB and LHG wrote the manuscript. SMB prepared the figures. All authors contributed to the article and approved the submitted version.

## Conflict of Interest

The authors declare no competing interests.

## AI declaration

We used ChatGPT (OpenAI; GPT‐5.3) during manuscript preparation to assist with language editing, clarity and text organisation. All scientific content and final wording were reviewed and approved by the authors. We take full responsibility for the content and accuracy of this work.

## Supporting information


**Table S1.** Overview of computational methods, required inputs, species limitations and accessibility.

**Table S2.** Curated list of case studies linking computational methods with corresponding experimental methods.

## Data Availability

Data sharing not applicable to this article as no datasets were generated or analysed during the current study.
