# Supplementary material for: Advances and opportunities for computational interrogation of plant proteins
Source: Plant J. 2026 Apr 29;126(3):e70899. doi: 10.1111/tpj.70899 (PMC13128292; doi:10.1111/tpj.70899)
Supplement: Supplementary file 1 — Table S1. Overview of computational methods, required inputs, species limitations and accessibility. [file TPJ-126-0-s001.pdf]

**Supporting Table 1. Overview of computational methods, required inputs, species limitations, and accessibility.** This table summarizes representative computational tools for ancestral sequence reconstruction (ASR), functional annotation, post-translational modification (PTM) prediction, localization prediction, protein structure and stability prediction, molecular dynamics (MD) simulations, and prediction of protein-protein, protein-nucleic-acid, and protein-metabolite interactions. For each method, the table lists key data inputs, typical data outputs, accessibility (e.g., webserver, source code, or software package), and organism specificity. Plant-specific and broadly applicable ("nonspecific") methods are indicated. Accessibility tiers are also indicated using symbols (rule of thumb for typical compute needs): Tier 1, open squares (web-based; minimal compute); Tier 2, hatched squares (local workstation or packaged distribution; moderate compute; often a single GPU is beneficial for batch jobs); and Tier 3, closed squares (HPC/GPU-intensive workflows, such as long all-atom MD simulations or local deep-learning structure prediction at scale). Multiple symbols indicate tools available through multiple access routes. Multiple tiers may also reflect scale dependence: workflows that are feasible locally for a single small protein can become time-prohibitive for large proteins, complexes, or datasets and for running long simulations or many replicate runs, motivating HPC/GPU use. Nonspecific methods can generally be applied across taxa, including plants, and many have already been successfully implemented in plant biology research. This compilation is not exhaustive but is intended as a practical reference for selecting computational tools based on available data types (e.g., single sequence, alignment, or structure).

| Method | Main Inputs | Main Outputs | Accessibility | Organisms | Reference |
|---|---|---|---|---|---|
| **Ancestral Sequence Reconstruction** | | | | | |
| ProtASR/ ProtASR2 | Sequence alignment<br>Phylogenetic tree<br>Protein structure | Reconstructed ancestral sequences<br>$\Delta G$ for different temperatures, residue pair mutation rates, residue contacts | Source code ▨■ | Nonspecific | Arenas et al., 2017<br>Arenas et al., 2020 |
| BetaAlign | Extant protein sequences | Protein sequence alignment | Source code ▨■ | Nonspecific | Dotan et al., 2025 |
| Phyloformer | Alignment | Phylogenetic tree | Source code ▨■ | Nonspecific | Nesterenko et al., 2025 |
| FastML | Alignment<br>Phylogenetic tree | Reconstructed ancestral sequences<br>Ancestral indels | Webserver □ | Nonspecific | Ashkenazy et al., 2012 |
| FireProtASR | Single protein sequence OR<br>Alignment OR<br>Phylogenetic tree | Reconstructed ancestral sequences | Webserver □<br>Source code ▨■ | Nonspecific | Musil et al., 2021 |
| IQTree | Alignment<br>Optional: Substitution Model | (Model selection)<br>Best-fitting substitution model<br>(Tree Inference)<br>Maximum likelihood phylogenetic tree | Webserver □<br>Source code ▨■ | Nonspecific | Nguyen et al., 2015<br>Trifinopoulos et al., 2016<br>Minh et al., 2020<br>Wong et al., 2025 |
| ModelFinder | Alignment | Best-fitting substitution model | Webserver (IQTree) □<br>Source code (IQTree) ▨■ | Nonspecific | Kalyaanamoorthy et al., 2017 |
| **Functional Annotation** | | | | | |
| Ensembl Variant Effect Predictor | Protein Variants<br>Selected genome | Deleteriousness scores<br>Variant frequencies<br>Phenotypes<br>Consequences per transcript | Webserver □<br>Source code ▨■ | Nonspecific | McLaren et al., 2016 |
| ANNOVAR | Protein Variants<br>Genome | Gene-based functional annotations | Webserver □<br>Source code ▨■ | Nonspecific | Wang et al., 2010 |

| | | | | | |
|---|---|---|---|---|---|
| SnpEff | Protein Variants<br>Selected reference database | Annotated variants | Source code ▨■ | Nonspecific | Cingolani et al., 2012 |
| MAGMA | GWAS statistics OR Genotypes/gene sets OR Reference linkage disequilibrium matrix | Predicted function (gene-property with p-values) | Source code ▨■ | Nonspecific | de Leeuw et al., 2015 |
| MASH | Matrix of observed variant effects AND Standard error OR Z-score matrix | Cis expression quantitative trait loci (eQTLs)<br>Effect estimates | R package ▨■ | Human | Urbut et al., 2019 |
| WGSA | Protein Variants | Annotated variants (including deleteriousness, frequency, conservation) | Source code ▨■<br>Amazon Machine Image ■ | Human | Liu et al., 2016 |
| PROVEAN | Protein sequence<br>Variants (single/list) | Annotated variants (classifications) | Source code ▨■ | Nonspecific | Choi et al., 2012 |
| LIST-S2 | Protein sequence<br>Mutations (single/list) | Deleteriousness score | Webserver □<br>Source code ▨■ | Nonspecific | Malhis et al., 2020 |
| SIFT | Protein variant sequence<br>Protein context | Deleteriousness score | Webserver □ | 204 Genomes | Kumar et al., 2009 |
| PolyPhen-2 | Protein variant sequence | Deleteriousness score | Webserver □<br>Source code ▨■ | Human | Adzhubei et al., 2010 |
| DeepEC-Transformer | Protein variant sequences (open reading frames) | Predicted enzyme commission (EC) # | Source code ▨■ | *E. coli*/<br>Bacteria | Kim et al., 2023 |
| DeepGOPlus | Protein sequence(s) | Gene ontology (Cellular Component, Molecular Function, and Biological Process with confidence scores)<br>Similar sequences with experimentally determined functions | Webserver □<br>Source code ▨■<br>REST API ▨■<br>SPARQL endpoint ▨■ | Nonspecific | Kulmanov & Hoehndorf, 2020 |
| DeepFRI | Protein sequence(s) OR Protein structure(s) | Gene ontology (Molecular Function, Biological Process, Cellular Component, and Enzyme Commission with probabilities, sequence/structure overlay and ontology explorer) | Webserver □<br>Source code ▨■ | Nonspecific | Gligorijević et al., 2021 |
| OpenCRAVAT | Protein variants | Variant assignments (inactivating, missense, non-silent)<br>Gene ontology<br>Cancer genome landscape assignments<br>Predicted domains<br>Annotator scores | Webserver □<br>Source code ▨■ | Human | Pagel et al., 2020 |
| easyGWAS | Select species and dataset<br>Phenotype(s)<br>SNPs or chromosomes | Association statistics | Webserver □<br>REST API ▨■ | Nonspecific | Grimm et al., 2017 |

| | | | | | |
|---|---|---|---|---|---|
| **Post-Translational Modification Prediction** | | | | | |
| NetPhos3.1 | Protein sequence(s) | Predicted phosphorylation sites | Webserver ☐ | Eukaryotes | Blom et al., 1999<br>Blom et al., 2004 |
| GPS 6.0 | Protein sequence(s)<br>Specific kinase family | Predicted phosphorylation sites | Webserver ☐<br>Source code ▨■ | Eukaryotes | Chen et al., 2023 |
| Phosformer-ST/<br>Phosformer | Protein sequence<br>Kinase sequence | Predicted kinase-specific phosphorylation sites | Webserver ☐<br>Source code ▨■ | Nonspecific/<br>Human | Zhou et al., 2023<br>Zhou et al., 2024 |
| MuSiteDeep | Protein sequence | Predicted phosphorylation sites<br>Sites mapped onto sequence and 3D structure | Webserver ☐<br>Source code ▨■ | Nonspecific | Wang et al., 2017<br>Wang et al., 2019<br>Wang et al., 2020 |
| StackGlyEmbed | Protein sequence | Predicted N-glycosylation sites<br>(N-X-[S-T]) | Source code ▨■ | Eukaryotes | Nafi & Rahman, 2025 |
| DeepNGlyPred | Protein sequence | Predicted N-glycosylation sites<br>(N-X-[S-T]) | Source code ▨■ | Human | Pakhrin et al., 2021 |
| EMNGly | Protein sequence(s) | Predicted N-glycosylation sites<br>(N-X-[S-T]) | Source code ▨■ | Nonspecific | Hou et al., 2023 |
| NetNGlyc | Protein sequence(s) | Predicted N-glycosylation sites<br>(N-X-[S-T] or all N) | Webserver ☐ | Human | Gupta & Brunak, 2002 |
| UbPred | Protein sequence | Predicted ubiquitinylated lysines | Webserver ☐<br>Source code ▨■ | Nonspecific | Radivojac et al., 2010 |
| UPFPSR | Protein sequence(s) | Predicted ubiquitinylated lysines | Source code ▨■ | Plants | Yin et al., 2022 |
| Ubigo-X | Protein sequence(s) | Predicted ubiquitination sites | Webserver ☐ | Nonspecific | Tantoh et al., 2025 |
| **Localization Prediction** | | | | | |
| WoLF PSORT | Protein sequence | Predicted subcellular localization | Webserver ☐<br>Wrappers ▨■ | Animal, Plant, Fungi | Horton et al., 2007 |
| DeepLoc 2.1 | Protein sequence | Predicted subcellular localization<br>Predicted membrane association | Webserver ☐<br>Source code ▨■ | Eukaryotes | Ødum et al., 2024<br>Nielsen 2025 |
| LocPro | Protein sequence | Predicted subcellular localization | Webserver ☐ | Nonspecific | Zhang et al., 2025 |
| LOCALIZER | Effector protein sequence | Predicted organelle localization | Webserver ☐<br>Source code ▨■ | Plants & Eukaryotes | Sperschneider et al., 2017 |
| Plant-mSubP | Protein sequence(s) | Predicted subcellular localization | Webserver ☐ | Plants | Sahu et al., 2020 |
| deepGPS/<br>openGPS | Protein sequence | Predicted subcellular localization<br>Fluorescence image predictions | Webserver ☐ | Human | Yuan et al., 2025 |
| PUPS | Protein sequence<br>Landmark stain images | Predicted subcellular localization at single-cell resolution<br>Predicted cellular images | Source code ▨■ | Human | Zhang et al., 2025 |

| | | | | | |
|---|---|---|---|---|---|
| **Protein Structure Prediction** | | | | | |
| MODELLER | Protein sequence<br>Template structure<br>Target-template alignment | Predicted protein structure | Source code ▨ ■ | Nonspecific | Webb & Sali 2016 |
| RosettaCM | Protein sequence<br>Template structure(s)<br>Target-template alignment | Homology models | Source code ▨ ■ | Nonspecific | Song et al., 2013 |
| I-TASSER | Protein sequence | Predicted protein structures<br>Functional annotation | Webserver ☐ | Nonspecific | Yang et al., 2015 |
| Phyre2 | Protein sequence | Predicted protein structure<br>Secondary structure, disorder, domains<br>Alignment | Webserver ☐ | Nonspecific | Kelley et al., 2015 |
| HHpred | Protein sequence(s) OR Alignment | Template hits<br>Target-template alignments<br>Predicted protein structure (generated with MODELLER) | Webserver ☐ | Select Archaeal, Bacterial, and Eukaryotic Proteomes | Zimmermann et al., 2018 |
| SWISS-MODEL | Protein sequence AND Target-template alignment AND/OR Template structure | Template hits<br>Homology models | Webserver ☐ | Nonspecific | Waterhouse et al., 2018 |
| LOMETS3 | Protein sequence (single chain) | Predicted structure<br>Solvent accessibility<br>Spatial restraints<br>Predicted domains<br>Template hits<br>Target-template alignments<br>Functional annotation | Webserver ☐ | Nonspecific | Zheng et al., 2022 |
| I-TASSER-MTD | Protein sequence | Predicted protein structure<br>Template hits<br>Secondary structure, domains<br>Solvent accessibility per residue<br>Functional annotation | Webserver ☐<br>Source code ▨ ■ | Nonspecific | Zhou et al., 2022 |
| Foldseek | Protein structure(s) OR Protein sequence | Structure search/Template hits<br>3D Alignments | Webserver ☐<br>Source code ▨ ■ | Nonspecific | van Kempen et al., 2024 |
| AlphaFold2 | Protein sequence | Predicted protein structure | Webserver ☐<br>Source code ▨ ■ | Nonspecific | Jumper et al., 2021 |
| AlphaFold (AF)-Multimer | Protein sequence(s) | Predicted protein structure | Webserver ☐<br>Source code ▨ ■ | Nonspecific | Evans et al., 2022 |
| AlphaFold3 | Protein sequences<br>Optional: Ion(s)<br>Nucleic acid sequence<br>Ligand sequence/identity | Predicted protein structure | Webserver ☐<br>Source code ▨ ■ | Nonspecific | Abramson et al., 2024 |

| | | | | | |
|---|---|---|---|---|---|
| RoseTTAFold | Protein sequence | Predicted protein structure<br>Predicted domains<br>Predicted disorder<br>Predicted transmembrane residues | Webserver ☐<br>Source code ▨ ■ | Nonspecific | Baek et al., 2021 |
| OpenFold | Protein sequence(s) | Predicted protein structure<br>Alignments | Source code ▨ ■ | Nonspecific | Ahdritz et al., 2024 |
| ESMFold | Protein sequence(s) | Predicted protein structure<br>Residue contacts | Webserver ☐<br>Source code ▨ ■ | Nonspecific | Lin et al., 2023 |
| OmegaFold | Single protein sequence | Predicted protein structure | Source code ▨ ■ | Nonspecific | Wu et al., 2022 |
| MULTICOM + AF-Multimer | Protein sequences | Protein complex models | Source code ▨ ■ | Nonspecific | Liu et al., 2023 |
| Phyre2.2 | Protein sequence | Predicted protein structure<br>Template hits<br>Alignment<br>Secondary structure, disorder, domains | Webserver ☐ | Nonspecific | Powell et al., 2025 |
| D-I-TASSER | Protein sequence | Predicted protein structure<br>Functional annotation | Webserver ☐ | Nonspecific | Zheng et al., 2025 |
| MoDAFold | Protein sequence | Predicted protein structure | Scripts ▨ ■ | Nonspecific | Zheng et al., 2024 |
| **Protein-Protein Interaction Prediction** | | | | | |
| HADDOCK/ HADDOCK 2.4 | Molecule structure(s)<br>Molecule type: protein, DNA, RNA, glycan, small molecule | Docked complex models<br>Interaction clusters<br>Intermolecular and interface statistics | Webserver ☐<br>Source code ▨ ■ | Nonspecific | Dominguez et al., 2003<br>Karaca et al., 2010<br>Honorato et al., 2021<br>Honorato et al., 2024 |
| RosettaDock | Protein structures | Docked complex models<br>Interface score | Webserver (ROSIE) ☐<br>Source code (Rosetta) ▨ ■ | Nonspecific | Lyskov & Gray 2008 |
| GREMLIN | Alignment for protein family | Protein contact scores | Source code ▨ ■ | Nonspecific | Balakrishnan et al., 2011 |
| plmDCA | Alignment | Contact map<br>(direct protein interactions/ couplings) | Source code ▨ ■ | Nonspecific | Ekeberg et al., 2014 |
| CCMpred | Alignment | Protein contacts | Source code ▨ ■ | Nonspecific | Seemayer et al., 2014 |
| RoseTTAFold2-PPI | Alignment | Residue interaction probabilities | Source code ▨ ■ | Nonspecific | Zhang et al., 2024 |
| ZDOCK | Protein structures | Docked complex models | Webserver ☐<br>Source code ▨ ■ | Nonspecific | Chen & Weng, 2003<br>Pierce et al., 2014 |
| ZEPPI | PPI structural models | Alignments<br>Interface scores | Source code ▨ ■ | Nonspecific | Zhao et al., 2024 |
| PPIP | Protein sequences | Predicted protein-protein interactions | Contact authors | Plants | Ding & Kihara, 2019 |

| | | | | | |
|---|---|---|---|---|---|
| DWPPI | Protein sequences | Predicted protein-protein interactions | Contact authors | Plants | Pan et al., 2022 |
| ESMAraPPI | Protein sequences | Predicted interaction scores | Source code | *Arabidopsis thaliana* | Zhou et al., 2023 |
| AraPathogen2.0 | Arabidopsis protein sequence(s) or proteome<br>Pathogen effector sequence(s) | Protein-pathogen interaction scores | Webserver<br>Source code | *Arabidopsis thaliana* | Lei et al., 2024 |
| PlantPathoPPI | Plant protein sequence(s)<br>Pathogen protein sequence(s) | Interaction prediction<br>(binary classification) | Webserver | Plants | Murmu et al., 2025 |
| MFGAC-PPI | Plant and pathogen protein sequences and structures | Predicted protein-pathogen interactions<br>(binary classification) | Source code | Plants | Wang et al., 2024 |
| AlphaPullDown2 | Protein sequence(s) | Predicted protein complex models | Source code | Nonspecific | Molodenskiy et al., 2025 |
| **Protein-Nucleic Acid Interaction Prediction** | | | | | |
| RoseTTAFoldNA | Protein sequence<br>Nucleic acid sequence<br>(RNA, dsDNA, ssDNA) | Predicted protein-NA complex<br>Distance distributions | Source code | Nonspecific | Baek et al., 2023 |
| DNAgenie | Protein sequence | Per-residue binary call and binding propensity for A-DNA, B-DNA, ssDNA | Webserver | Nonspecific | Zhang et al., 2021 |
| iDRNA-ITF | Protein sequence | Per-residue binding probability for DNA, RNA | Webserver | Nonspecific | Wang et al., 2022 |
| PlantBind | DNA sequence<br>DNA shape feature file | Predicted transcription factor binding sites | Webserver<br>Source code | *Arabidopsis thaliana* | Yan et al., 2022 |
| SeqConv | TF ChIP-seq peak file<br>Genome | Transcription factor binding probabilities | Source code | Plants | Shen et al., 2021 |
| TSPTFBS2.0/<br>TSPTFBS | DNA sequence<br>Selected TF model | Transcription factor binding probabilities | Webserver<br>Source code<br>Docker imager | *Arabidopsis thaliana; v2.0: + Zea mays Orzya sativa* | Liu et al., 2021<br>Cheng et al., 2023 |
| PTFSpot | DNA sequences<br>Protein structure | TF binding region of DNA | Webserver<br>Source code | Plants | Gupta et al., 2024 |
| HDOCK | Protein and nucleic acid structure(s) or sequence(s) | Predicted complex structures | Webserver | Nonspecific | Yan et al., 2017<br>Yan et al., 2020 |
| NPDOCK | Protein structure<br>Nucleic acid structure | Predicted complex structures | Webserver | Nonspecific | Tuszynska et al., 2015 |
| DeepDISOBind | Protein sequence(s) | Per-residue binding probability for protein, DNA, RNA (binding to disordered regions) | Webserver | Nonspecific | Zhang et al., 2022 |

| | | | | | |
|---|---|---|---|---|---|
| Plant-DTI | Plant TF sequence<br>Promoter DNA sequence | Predicted TF-promoter interactions | Webserver □<br>Source code ▨ ■ | Plants | Ruengsrichaiya et al., 2022 |
| **Protein-Metabolite Interaction Prediction** | | | | | |
| ConCavity | Protein structure | Per-residue metabolite binding propensity | Webserver □<br>Source code ▨ ■ | Nonspecific | Capra et al., 2009 |
| Patch-Surfer2.0/<br>PL-PatchSurfer2 | Binding pocket structure<br>Ligand structures | Metabolite/Ligand hits<br>(ranked by binding site similarity) | Source code ▨ ■ | Nonspecific | Zhu et al., 2015<br>Shin et al., 2016 |
| CurPocket<br>(Algorithm used in CB-Dock/2) | Protein structure | Predicted binding pockets | Webserver (CB-Dock/2) □ | Nonspecific | Liu et al., 2020 |
| CB-Dock2 | Protein structure<br>Ligand structures | Predicted cavities/binding pockets<br>Binding poses and contact residues<br>(structure and template-based detection) | Webserver □ | Nonspecific | Liu et al., 2022 |
| DeepPocket | Protein structure | Predicted binding pockets | Source code ▨ ■ | Nonspecific | Aggarwal et al., 2022 |
| Kalasanty | Protein structure | Predicted binding pockets<br>Volumetric density maps | Source code ▨ ■ | Nonspecific | Stepniewska-Dziubinska et al., 2020 |
| AutoDock Vina (1.2.0) | Receptor protein structure<br>Ligand structure | Docking conformations<br>(Ligand orientation/conformation) | Source code ▨ ■ | Nonspecific | Eberhardt et al., 2021 |
| GNINA1.0 | Receptor protein structure<br>Ligand structure | Docked conformations<br>(Ligand orientation/conformation) | Source code ▨ ■ | Nonspecific | McNutt et al., 2021 |
| Vina-GPU 2.1 | Receptor protein structure<br>Ligand structure | Docked conformations<br>(Ligand orientation/conformation) | Source code ▨ ■ | Nonspecific | Tang et al., 2024 |
| HNCGAT | Proteins, Metabolites, Functional Annotations (from databases) | Predicted protein-metabolite interactions | Source code ▨ ■ | *Arabidopsis thaliana* | Zhou et al., 2024 |
| **Protein Stability Prediction** | | | | | |
| FoldX | Protein structure<br>Mutation list | Change in folding free energy upon mutation (kcal/mol) | Source code ▨ ■ | Nonspecific | Delgado et al., 2019<br>Delgado et al., 2025 |
| Rosetta ddG_monomer | Protein structure<br>Mutation list | Change in folding free energy upon mutation (REU) | Source code (Rosetta) ▨ ■ | Nonspecific | Kellogg et al., 2011 |
| Rosetta cartesian_ddG | Protein structure<br>Mutations list | Change in folding free energy upon mutation (REU) | Source code (Rosetta) ▨ ■ | Nonspecific | Park et al., 2016<br>Frenz et al., 2020 |
| Rosetta flex ddG | Protein structure<br>Mutation list | Interface $\Delta\Delta G$ upon mutation (REU) | Source code ▨ ■ | Nonspecific | Barlow et al., 2018 |
| DynaMut/<br>DynaMut2 | Protein structure AND<br>For Mutation Predictions: Mutation (single, list)<br>For Normal Mode Analysis: Force Field | Deformation energies<br>Atomic fluctuations<br>Residue interactions<br>For Mutation Predictions:<br>$\Delta\Delta G_{folding}$ (kcal/mol), $\Delta\Delta S_{vibrational}$ | Webserver □ | Nonspecific | Rodrigues et al., 2018<br>Rodrigues et al., 2021 |

| | | | | | |
|---|---|---|---|---|---|
| | | For Normal Mode Analysis: Predicted molecular motion, cross-correlation of residues | | | |
| CNAnalysis | Protein structure OR Structure ensemble networks | Global and local flexibility/rigidity<br>Rigidity order and cluster configuration<br>Entropy transitions<br>Stability map of residue contacts<br>Unfolding nuclei | Webserver □<br>Software ▨ ■ | Nonspecific | Pfleger et al., 2013<br>Krüger et al., 2013 |
| WebPSN/ PSN | Protein or Nucleic acid structures OR MD trajectory files | Structure networks<br>Metapaths<br>Correlated residues | Webserver □<br>Source code ▨ ■ | Nonspecific | Felline et al., 2022 |
| ThermoNet | Protein structure<br>Mutations (single/list) | Change in folding free energy upon mutation (kcal/mol) | Source code ▨ ■ | Nonspecific | Li et al., 2020 |
| DDMut | Protein structure AND For Mutation Predictions: Mutations (single/list/alanine scanning)<br>For Normal Mode Analysis:<br>Force field | Mutation Predictions: forward and reverse predicted $\Delta\Delta G_{folding}$ (kcal/mol)<br>Normal Mode Analysis: Predicted molecular motion, cross-correlation of residues | Webserver □ | Nonspecific | Zhou et al., 2023 |
| DDGemb | Protein sequence<br>Mutations (single/list) | Change in folding free energy upon mutation | Webserver □ | Nonspecific | Savojardo et al., 2025 |
| RaSP | Protein structure<br>Mutations (single/list) | Change in folding free energy upon mutation (kcal/mol) | Colab notebook □<br>Source code ▨ ■ | Nonspecific | Blaabjerg et al., 2023 |
| MSA Transformer | Alignment | Per-sequence representations<br>(can be used for contact prediction and structure prediction) | Source code ▨ ■ | Nonspecific | Rao et al., 2021 |
| PremPS | Protein structure<br>Mutations (single/alanine scanning) | Change in folding free energy upon mutation (kcal/mol) | Webserver □<br>Source code ▨ ■ | Nonspecific | Chen et al., 2020 |
| MAESTRO/ MAESTROweb | Protein structure AND Evaluate mutations (single/list) OR generate mutation sensitivity profile OR scan for stabilizing or destabilizing mutations OR identify potential disulfide bonds | (Mutations)<br>per-mutation $\Delta\Delta G$ (kcal/mol)<br>(Sensitivity Profile)<br>per-residue $\Delta\Delta G$ (kcal/mol)<br>(Disulfide Bonds)<br>per-bond $\Delta\Delta G$ (kcal/mol), bond scores | Webserver □<br>Source code ▨ ■ | Nonspecific | Laimer et al., 2015 |

| **Molecular Dynamics Simulation** | | | | | |
|---|---|---|---|---|---|
| WebGRO/ GROMACS | Atomic coordinates<br>Topology & parameters/ Protein structure<br>Specified force field | MD trajectories | Webserver □<br>Source code ▨ ■ | Nonspecific | Páll et al., 2020<br>Abraham et al., 2015<br>Páll et al., 2015<br>Pronk et al., 2013<br>Hess et al., 2008<br>van der Spoel et al., 2005<br>Lindahl et al., 2001<br>Berendsen et al., 1995 |
| AMBER | Atomic coordinates<br>Topology & parameters | MD trajectories | Source code ▨ ■ | Nonspecific | Case et al., 2005<br>Case et al., 2023 |
| DL_POLY | Atomic coordinates<br>Topology & parameters | MD trajectories | Source code ▨ ■ | Nonspecific | Smith et al., 2002<br>Devereux et al., 2025 |
| NAMD | Protein structure(s)<br>Atomic coordinates file<br>Force field parameters | MD trajectories | Source code ▨ ■ | Nonspecific | Phillips et al., 2005<br>Phillips et al., 2020 |
| GENESIS 2.1 | Protein structure(s)<br>Topology & parameters | MD trajectories | Source code ▨ ■ | Nonspecific | Jung et al., 2024 |
| DeepDriveMD | Initial MD trajectories | New MD trajectories | Source code ▨ ■ | Nonspecific | Lee et al., 2019 |
| TorchMD-NET | Atomic positions<br>Embedding Indices<br>(incl. atomic #s) | Predicted energies<br>(per-molecule energy values, often taken to be potential energy) | Source code ▨ ■ | Nonspecific | Thölke & De Fabritiis, 2022<br>Pelaez et al., 2024 |

## Supporting Table 1 References


Abraham, M.J., Murtola, T., Schulz, R., Páll, S., Smith, J.C., Hess, B., Lindahl, E., 2015. GROMACS: High performance molecular simulations through multi-level parallelism from laptops to supercomputers. SoftwareX 1–2, 19–25. https://doi.org/10.1016/j.softx.2015.06.001

Ahdritz, G., Bouatta, N., Floristean, C., Kadyan, S., Xia, Q., Gerecke, W., O'Donnell, T.J., Berenberg, D., Fisk, I., Zanichelli, N., Zhang, B., Nowaczynski, A., Wang, B., Stepniewska-Dziubinska, M.M., Zhang, S., Ojewole, A., Guney, M.E., Biderman, S., Watkins, A.M., Ra, S., Lorenzo, P.R., Nivon, L., Weitzner, B., Ban, Y.-E.A., Chen, S., Zhang, M., Li, C., Song, S.L., He, Y., Sorger, P.K., Mostaque, E., Zhang, Z., Bonneau, R., AlQuraishi, M., 2024. OpenFold: retraining AlphaFold2 yields new insights into its learning mechanisms and capacity for generalization. Nat Methods 21, 1514–1524. https://doi.org/10.1038/s41592-024-02272-z

Balakrishnan, S., Kamisetty, H., Carbonell, J.G., Lee, S.-I., Langmead, C.J., 2011. Learning generative models for protein fold families. Proteins: Structure, Function, and Bioinformatics 79, 1061–1078. https://doi.org/10.1002/prot.22934

Berendsen, H.J.C., van der Spoel, D., van Drunen, R., 1995. GROMACS: A message-passing parallel molecular dynamics implementation. Computer Physics Communications 91, 43–56. https://doi.org/10.1016/0010-4655(95)00042-E

Blom, N., Gammeltoft, S., Brunak, S., 1999. Sequence and structure-based prediction of eukaryotic protein phosphorylation sites. J Mol Biol 294, 1351–1362. https://doi.org/10.1006/jmbi.1999.3310

Blom, N., Sicheritz-Pontén, T., Gupta, R., Gammeltoft, S., Brunak, S., 2004. Prediction of post-translational glycosylation and phosphorylation of proteins from the amino acid sequence. Proteomics 4, 1633–1649. https://doi.org/10.1002/pmic.200300771

Chen, M., Zhang, W., Gou, Y., Xu, D., Wei, Y., Liu, D., Han, C., Huang, X., Li, C., Ning, W., Peng, D., Xue, Y., 2023. GPS 6.0: an updated server for prediction of kinase-specific phosphorylation sites in proteins. Nucleic Acids Res 51, W243–W250. https://doi.org/10.1093/nar/gkad383

Chen, R., Li, L., Weng, Z., 2003. ZDOCK: an initial-stage protein-docking algorithm. Proteins 52, 80–87. https://doi.org/10.1002/prot.10389

Cheng, H., Liu, L., Zhou, Y., Deng, K., Ge, Y., Hu, X., 2023. TSPTFBS 2.0: trans-species prediction of transcription factor binding sites and identification of their core motifs in plants. Front. Plant Sci. 14. https://doi.org/10.3389/fpls.2023.1175837

Choi, Y., Sims, G.E., Murphy, S., Miller, J.R., Chan, A.P., 2012. Predicting the functional effect of amino acid substitutions and indels. PLoS One 7, e46688. https://doi.org/10.1371/journal.pone.0046688

Delgado, J., Reche, R., Cianferoni, D., Orlando, G., van der Kant, R., Rousseau, F., Schymkowitz, J., Serrano, L., 2025. FoldX force field revisited, an improved version. Bioinformatics 41, btaf064. https://doi.org/10.1093/bioinformatics/btaf064

Devereux, H.L., Cockrell, C., Elena, A.M., Bush, I., Chalk, A.B.G., Madge, J., Scivetti, I., Wilkins, J.S., Todorov, I.T., Smith, W., Trachenko, K., 2025. arXiv [preprint]. DL_POLY 5: Calculation of system properties on the fly for very large systems via massive parallelism. https://doi.org/10.48550/arXiv.2503.07526

Dominguez, C., Boelens, R., Bonvin, A.M.J.J., 2003. HADDOCK:  A Protein−Protein Docking Approach Based on Biochemical or Biophysical Information. J. Am. Chem. Soc. 125, 1731–1737. https://doi.org/10.1021/ja026939x

Grimm, D.G., Roqueiro, D., Salomé, P.A., Kleeberger, S., Greshake, B., Zhu, W., Liu, C., Lippert, C., Stegle, O., Schölkopf, B., Weigel, D., Borgwardt, K.M., 2017. easyGWAS: A Cloud-Based Platform for Comparing the Results of Genome-Wide Association Studies. Plant Cell 29, 5–19. https://doi.org/10.1105/tpc.16.00551

Hess, B., Kutzner, C., van der Spoel, D., Lindahl, E., 2008. GROMACS 4: Algorithms for Highly Efficient, Load-Balanced, and Scalable Molecular Simulation. J. Chem. Theory Comput. 4, 435–447. https://doi.org/10.1021/ct700301q

Honorato, R.V., Koukos, P.I., Jiménez-García, B., Tsaregorodtsev, A., Verlato, M., Giachetti, A., Rosato, A., Bonvin, A.M.J.J., 2021. Structural Biology in the Clouds: The WeNMR-EOSC Ecosystem. Front. Mol. Biosci. 8. https://doi.org/10.3389/fmolb.2021.729513

Karaca, E., Melquiond, A.S.J., Vries, S.J. de, Kastritis, P.L., Bonvin, A.M.J.J., 2010. Building Macromolecular Assemblies by Information-driven Docking. Molecular & Cellular Proteomics 9, 1784–1794. https://doi.org/10.1074/mcp.M000051-MCP201

Kelley, L.A., Mezulis, S., Yates, C.M., Wass, M.N., Sternberg, M.J.E., 2015. The Phyre2 web portal for protein modeling, prediction and analysis. Nat Protoc 10, 845–858. https://doi.org/10.1038/nprot.2015.053

Lei, C., Zhou, K., Zheng, J., Zhao, M., Huang, Y., He, H., Yang, S., Zhang, Z., 2024. AraPathogen2.0: An Improved Prediction of Plant-Pathogen Protein-Protein Interactions Empowered by the Natural Language Processing Technique. J Proteome Res 23, 494–499. https://doi.org/10.1021/acs.jproteome.3c00364

Li, B., Yang, Y.T., Capra, J.A., Gerstein, M.B., 2020. Predicting changes in protein thermodynamic stability upon point mutation with deep 3D convolutional neural networks. PLoS Comput Biol 16, e1008291. https://doi.org/10.1371/journal.pcbi.1008291

Lindahl, E., Hess, B., van der Spoel, D., 2001. GROMACS 3.0: a package for molecular simulation and trajectory analysis. J Mol Model 7, 306–317. https://doi.org/10.1007/s008940100045

Liu, J., Guo, Z., Wu, T., Roy, R.S., Quadir, F., Chen, C., Cheng, J., 2023. Enhancing alphafold-multimer-based protein complex structure prediction with MULTICOM in CASP15. Commun Biol 6, 1140. https://doi.org/10.1038/s42003-023-05525-3

Liu, Y., Yang, X., Gan, J., Chen, S., Xiao, Z.-X., Cao, Y., 2022. CB-Dock2: improved protein–ligand blind docking by integrating cavity detection, docking and homologous template fitting. Nucleic Acids Res 50, W159–W164. https://doi.org/10.1093/nar/gkac394

Malhis, N., Jacobson, M., Jones, S.J.M., Gsponer, J., 2020. LIST-S2: taxonomy based sorting of deleterious missense mutations across species. Nucleic Acids Res 48, W154–W161. https://doi.org/10.1093/nar/gkaa288

McLaren, W., Gil, L., Hunt, S.E., Riat, H.S., Ritchie, G.R.S., Thormann, A., Flicek, P., Cunningham, F., 2016. The Ensembl Variant Effect Predictor. Genome Biol 17, 122. https://doi.org/10.1186/s13059-016-0974-4

Minh, B.Q., Schmidt, H.A., Chernomor, O., Schrempf, D., Woodhams, M.D., von Haeseler, A., Lanfear, R., 2020. IQ-TREE 2: New Models and Efficient Methods for Phylogenetic Inference in the Genomic Era. Mol Biol Evol 37, 1530–1534. https://doi.org/10.1093/molbev/msaa015

Nielsen, H., 2025. Practical Applications of Language Models in Protein Sorting Prediction: SignalP 6.0, DeepLoc 2.1, and DeepLocPro 1.0, in: KC, D.B. (Ed.), Large Language Models (LLMs) in Protein Bioinformatics. Springer US, New York, NY, pp. 153–175. https://doi.org/10.1007/978-1-0716-4623-6_10

Páll, S., Abraham, M.J., Kutzner, C., Hess, B., Lindahl, E., 2015. Tackling Exascale Software Challenges in Molecular Dynamics Simulations with GROMACS, in: Markidis, S., Laure, E. (Eds.), Solving Software Challenges for Exascale. Springer International Publishing, Cham, pp. 3–27. https://doi.org/10.1007/978-3-319-15976-8_1

Park, H., Bradley, P., Greisen, P.Jr., Liu, Y., Mulligan, V.K., Kim, D.E., Baker, D., DiMaio, F., 2016. Simultaneous Optimization of Biomolecular Energy Functions on Features from Small Molecules and Macromolecules. J. Chem. Theory Comput. 12, 6201–6212. https://doi.org/10.1021/acs.jctc.6b00819

Pan, J., You, Z.-H., Li, L.-P., Huang, W.-Z., Guo, J.-X., Yu, C.-Q., Wang, L.-P., Zhao, Z.-Y., 2022. DWPPI: A Deep Learning Approach for Predicting Protein–Protein Interactions in Plants Based on Multi-Source Information With a Large-Scale Biological Network. Front. Bioeng. Biotechnol. 10. https://doi.org/10.3389/fbioe.2022.807522

Phillips, J.C., Braun, R., Wang, W., Gumbart, J., Tajkhorshid, E., Villa, E., Chipot, C., Skeel, R.D., Kalé, L., Schulten, K., 2005. Scalable molecular dynamics with NAMD. J Comput Chem 26, 1781–1802. https://doi.org/10.1002/jcc.20289

Phillips, J.C., Hardy, D.J., Maia, J.D.C., Stone, J.E., Ribeiro, J.V., Bernardi, R.C., Buch, R., Fiorin, G., Hénin, J., Jiang, W., McGreevy, R., Melo, M.C.R., Radak, B.K., Skeel, R.D., Singharoy, A., Wang, Y., Roux, B., Aksimentiev, A., Luthey-Schulten, Z., Kalé, L.V., Schulten, K., Chipot, C., Tajkhorshid, E., 2020. Scalable molecular dynamics on CPU and GPU architectures with NAMD. J Chem Phys 153, 044130. https://doi.org/10.1063/5.0014475

Pierce, B.G., Wiehe, K., Hwang, H., Kim, B.-H., Vreven, T., Weng, Z., 2014. ZDOCK server: interactive docking prediction of protein–protein complexes and symmetric multimers. Bioinformatics 30, 1771–1773. https://doi.org/10.1093/bioinformatics/btu097

Pronk, S., Páll, S., Schulz, R., Larsson, P., Bjelkmar, P., Apostolov, R., Shirts, M.R., Smith, J.C., Kasson, P.M., van der Spoel, D., Hess, B., Lindahl, E., 2013. GROMACS 4.5: a high-throughput and highly parallel open source molecular simulation toolkit. Bioinformatics 29, 845–854. https://doi.org/10.1093/bioinformatics/btt055

Rodrigues, C.H., Pires, D.E., Ascher, D.B., 2018. DynaMut: predicting the impact of mutations on protein conformation, flexibility and stability. Nucleic Acids Res 46, W350–W355. https://doi.org/10.1093/nar/gky300

Seemayer, S., Gruber, M., Söding, J., 2014. CCMpred—fast and precise prediction of protein residue–residue contacts from correlated mutations. Bioinformatics 30, 3128–3130. https://doi.org/10.1093/bioinformatics/btu500

Shin, W.-H., Christoffer, C.W., Wang, J., Kihara, D., 2016. PL-PatchSurfer2: Improved Local Surface Matching-Based Virtual Screening Method That Is Tolerant to Target and Ligand Structure Variation. J Chem Inf Model 56, 1676–1691. https://doi.org/10.1021/acs.jcim.6b00163

Song, Y., DiMaio, F., Wang, R.Y.-R., Kim, D., Miles, C., Brunette, T., Thompson, J., Baker, D., 2013. High resolution comparative modeling with RosettaCM. Structure 21, 10.1016/j.str.2013.08.005. https://doi.org/10.1016/j.str.2013.08.005

Tantoh, D.M., Yu, J.-C., Chien, C.-H., Yeh, W.-Y., Chu, Y.-W., 2025. Ubigo-X: Protein ubiquitination site prediction using ensemble learning with image-based feature representation and weighted voting. Computational and Structural Biotechnology Journal 27, 3137–3146. https://doi.org/10.1016/j.csbj.2025.07.025

Thölke, P., De Fabritiis, G., 2022. TorchMD-NET: Equivariant Transformers for Neural Network based Molecular Potentials. https://doi.org/10.48550/arXiv.2202.02541

Trifinopoulos, J., Nguyen, L.-T., von Haeseler, A., Minh, B.Q., 2016. W-IQ-TREE: a fast online phylogenetic tool for maximum likelihood analysis. Nucleic Acids Res 44, W232–W235. https://doi.org/10.1093/nar/gkw256

Van Der Spoel, D., Lindahl, E., Hess, B., Groenhof, G., Mark, A.E., Berendsen, H.J.C., 2005. GROMACS: Fast, flexible, and free. Journal of Computational Chemistry 26, 1701–1718. https://doi.org/10.1002/jcc.20291

Wang, D., Liang, Y., Xu, D., 2019. Capsule network for protein post-translational modification site prediction. Bioinformatics 35, 2386–2394. https://doi.org/10.1093/bioinformatics/bty977

Wang, D., Zeng, S., Xu, C., Qiu, W., Liang, Y., Joshi, T., Xu, D., 2017. MusiteDeep: a deep-learning framework for general and kinase-specific phosphorylation site prediction. Bioinformatics 33, 3909–3916. https://doi.org/10.1093/bioinformatics/btx496

Webb, B., Sali, A., 2016. Comparative Protein Structure Modeling Using MODELLER. Current Protocols in Bioinformatics 54, 5.6.1-5.6.37. https://doi.org/10.1002/cpbi.3

Yan, Y., Zhang, D., Zhou, P., Li, B., Huang, S.-Y., 2017. HDOCK: a web server for protein–protein and protein–DNA/RNA docking based on a hybrid strategy. Nucleic Acids Res 45, W365–W373. https://doi.org/10.1093/nar/gkx407

Yang, J., Yan, R., Roy, A., Xu, D., Poisson, J., Zhang, Y., 2015. The I-TASSER Suite: protein structure and function prediction. Nature Methods 12, 7–8. https://doi.org/10.1038/nmeth.3213

Zhang, J., Ghadermarzi, S., Katuwawala, A., Kurgan, L., 2021. DNAgenie: accurate prediction of DNA-type-specific binding residues in protein sequences. Brief Bioinform 22, bbab336. https://doi.org/10.1093/bib/bbab336

Zheng, W., Wuyun, Q., Zhou, X., Li, Y., Freddolino, L., Zhang, Y., 2022. LOMETS3: integrating deep learning and profile alignment for advanced protein template recognition and function annotation. Nucleic Acids Res 50, W454–W464. https://doi.org/10.1093/nar/gkac248

Zhou, Y., Pan, Q., Pires, D.E.V., Rodrigues, C.H.M., Ascher, D.B., 2023. DDMut: predicting effects of mutations on protein stability using deep learning. Nucleic Acids Res 51, W122–W128. https://doi.org/10.1093/nar/gkad472

Zhou, Z., Yeung, W., Soleymani, S., Gravel, N., Salcedo, M., Li, S., Kannan, N., 2024. Using explainable machine learning to uncover the kinase–substrate interaction landscape. Bioinformatics 40, btae033. https://doi.org/10.1093/bioinformatics/btae033
