# Supplementary material for: Advances and opportunities for computational interrogation of plant proteins
Source: Plant J. 2026 Apr 29;126(3):e70899. doi: 10.1111/tpj.70899 (PMC13128292; doi:10.1111/tpj.70899)
Supplement: Supplementary file 2 — Table S2. Curated list of case studies linking computational methods with corresponding experimental methods. [file TPJ-126-0-s002.pdf]

Supporting Table 2. Curated list of case studies linking computational methods with corresponding experimental methods. This table summarizes representative studies using computational approaches for ancestral sequence reconstruction (ASR); function, localisation, and post-translational modification (PTM) prediction; protein structure prediction; prediction of protein-protein, protein-nucleic acid, and protein-metabolite interactions; and stability and flexibility predictions. For each case study, the table lists a statement of purpose describing the biological question being studied, the utilized computational method, and the major corresponding experimental method(s), or other validation approaches, used to test the computational output or model. In studies lacking new experimental validation, as is common in method-focused papers, model validation strategies or agreement with previously published results are noted. For MD case studies in which experimental validation has not yet been completed, a potential future experimental validation approach is indicated. This curated subset of examples from the main text is intended as a reference for identifying experimental methods that can complement selected computational approaches. BiFC, bimolecular fluorescence complementation; SAXS, small-angle X-ray scattering; SEC-MALS, size-exclusion chromatography with multi-angle light scattering; Co-IP, co-immunoprecipitation; BN-PAGE, blue native polyacrylamide gel electrophoresis; PPI, protein–protein interaction; ChIP-seq, chromatin immunoprecipitation sequencing; DAP-seq, DNA affinity purification sequencing; Y1H, yeast one-hybrid; EDS, energy-dispersive X-ray spectroscopy; FTIR, Fourier transform infrared spectroscopy.

Study Purpose	Computational Method	Main Validation	Citation
Ancestral Sequence Reconstruction			
Study the evolutionary history of iridoid biosynthesis and its re-emergence in *Nepeta* (catnip)	FastML	Recombinant proteins:<br>*In vitro* enzyme activity assays	Lichman et al., 2020
Determine when pyrenoid-forming and carbon-concentrating functions emerged during EPYC1 evolution	IQ-TREE with PASTML for gap assignment	Recombinant ancestral and modern EPYC1 Rubisco fusion proteins:<br>*In vitro* phase-separation<br>Enzyme activity assays	Küffner et al., 2024
Determine when Rubisco became dependent on assembly chaperones during evolution, and whether that dependence was associated with improved catalytic function	PAML for RbcS, RbcL<br>IQ-TREE for Raf1 and RbcX with PASTML for gap assignment	Recombinant proteins:<br>Rubisco assembly assays<br>Enzyme activity assays	Ng et al., 2025
Function, Localisation, and Modification			
Identify the gene underlying a suppressor phenotype affecting ethylene-responsive seed germination in a *prt6* background	ANNOVAR	Phenotyping:<br>Ethylene sensitivity in CRISPR-Cas9 knockout seeds	Wang et al., 2025
Identify lysine residues in rice flowering repressor Ghd7 that undergo sucrose-induced polyubiquitination and degradation	UbPred	Mutant Ghd7 proteins:<br>Expression in presence or absence of sucrose to test degradation	Cho et al., 2024
Determine which *Physcomitrium patens* PpNBS1 domains mediate oxidative stress tolerance,	LOCALIZER	Truncation-mutant GFP fusion proteins:<br>Fluorescence microscopy	Das et al., 2025

localization, and MRE11 interaction			
Protein Structure Prediction			
Elucidate the mechanism of action for Arabidopsis phosphate transporter 1;1 (AtPHT1;1)	SWISS-MODEL	Mutant proteins:<br>Complementation assays<br>Phosphate transport activity assays<br>Mutant YFP fusion proteins:<br>Confocal microscopy	Liao et al., 2019
Generate mechanistic hypotheses for the regulation of starch degradation by α- and β-amylases	AlphaFold3	Recombinant proteins:<br>Enzyme activity assays<br>Crosslinking<br>SAXS/SEC-MALS	Berndsen et al., 2025
Identify a potential mechanism of action by which Cyclophilin 38 stabilises photosystem II through interaction with thylakoid lumen protein 7.6	AlphaFold3	Co-IP<br>CRISPR-Cas9 knockout proteins:<br>Photosynthetic phenotyping<br>Ultrastructure imaging<br>BN-PAGE	Ren et al., 2025
Modeling Protein Interactions (Protein-Protein, Protein-Nucleic Acid, Protein-Ligand/Metabolite)			
Identify plant-pathogen PPIs involved in pine immunity against Pine Wilt Disease	MFGAC-PPI	Benchmarking against known PPIs<br>No new experimental validation	Wang et al., 2024
Study mechanisms of regulation for cassava MeSUS1, a key sucrose metabolism enzyme	Plant-DTI	Benchmarking against ChIP-seq and DAP-seq datasets<br>Prediction agreement with prior Y1H validation<br>No new experimental validation	Ruengsrichaiya et al., 2022
Predict the guanine protein-metabolite network in Arabidopsis	HNCGAT	Benchmarking against known interactions and literature-supported case studies<br>No new experimental validation	Zhou et al., 2024
Protein Flexibility & Stability			
Engineer *Solanum tuberosum* patatin variants with improved thermostability	CNAnalysis	Mutant patatin proteins:<br>Thermal shift assays	Friberg et al., 2025
Identify transcriptional regulators that confer salinity tolerance in wheat via stable protein-DNA interactions	AMBER	No new experimental validation<br>Future experimentation: salinity stress phenotyping	Hassan et al., 2022
Identify rice PGPRs involved in pathogen defense against *Magnaporthe grisea*	GROMACS	MD trajectories/backbone deviation consistent with the predicted docked glucanase-GTP complex conformation<br>No new experimental validation<br>Future experimentation: GTP binding assays with glucanase mutants	Jha et al., 2022
